# Two New Phenylhydrazone Derivatives from the Pearl River Estuary Sediment-Derived *Streptomyces* sp. SCSIO 40020

**DOI:** 10.3390/md20070449

**Published:** 2022-07-09

**Authors:** Wei Liu, Liang Ma, Liping Zhang, Yuchan Chen, Qingbo Zhang, Haibo Zhang, Weimin Zhang, Changsheng Zhang, Wenjun Zhang

**Affiliations:** 1 Key Laboratory of Tropical Marine Bio-Resources and Ecology, Guangdong Key Laboratory of Marine Materia Medica, South China Sea Institute of Oceanology, Innovation Academy for South China Sea Ecology and Environmental Engineering, Chinese Academy of Sciences, 164 West Xingang Road, Guangzhou 510301, China; wwl1430@outlook.com (W.L.); maliangyc@hotmail.com (L.M.); zhanglp@scsio.ac.cn (L.Z.); gudaobo@163.com (Q.Z.); zhanghb@scsio.ac.cn (H.Z.); 2 State Key Laboratory of Applied Microbiology Southern China, Institute of Microbiology, Guangdong Academy of Sciences, 100 Central Xianlie Road, Guangzhou 510070, China; chenyc@gdim.cn (Y.C.); wmzhang@gdim.cn (W.Z.); 3 Southern Marine Science and Engineering Guangdong Laboratory (Guangzhou), No.1119, Haibin Road, Guangzhou 511458, China; 4 University of Chinese Academy of Sciences, Beijing 100049, China

**Keywords:** penzonemycins, phenylhydrazones, *Streptomyces* sp., Japp–Klingemann coupling reaction, marine natural products

## Abstract

Two new phenylhydrazone derivatives and one new alkaloid, penzonemycins A–B (**1**–**2**) and demethylmycemycin A (**3**), together with three known compounds including an alkaloid (**4**) and two sesquiterpenoids (**5**–**6**), were isolated from the *Streptomyces* sp. SCSIO 40020 obtained from the Pearl River Estuary sediment. Their structures and absolute configurations were assigned by 1D/2D NMR, mass spectroscopy and X-ray crystallography. Compound **1** was evaluated in four human cancer cell lines by the SRB method and displayed weak cytotoxicity in three cancer cell lines, with IC_50_ values that ranged from 30.44 to 61.92 µM, which were comparable to those of the positive control cisplatin. Bioinformatic analysis of the putative biosynthetic gene cluster indicated a Japp–Klingemann coupling reaction involved in the hydrazone formation of **1** and **2**.

## 1. Introduction

Hydrazones are a class of compounds with the characteristic structure R_1_R_2_C=NNR_3_R_4_. Hydrazones display various bioactivities, including antimicrobial, antitubercular, anti-HIV, anti-inflammatory, anticonvulsant, analgesic and antitumor properties [1]. The unique chemical properties and bioactivities of hydrazones have inspired organic chemists to develop numerous methods for the synthesis of their derivatives [2,3]. In contrast to the large number of synthetic hydrazones, only a handful of naturally occurring hydrazones are found [4,5,6,7,8,9] (Figure S1). To date, 38 natural hydrazones have been reported, including 21 from fungi [5,9], 13 from actinomycetes [4,6,7,8], and 4 from other resources [9] (Figure S1).

**Figure 1:**
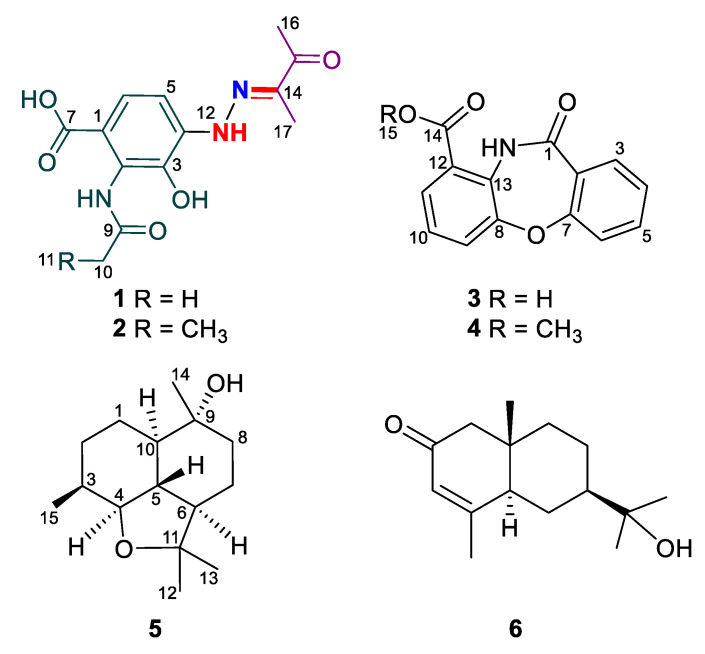
Chemical structures of compounds **1**–**6**.

Marine-derived actinomycetes have been a prolific resource for novel natural product discovery [10,11]. Previously, we reported three novel hydrazone derivatives, pyrazolofluostatins A–C, which were isolated from South China Sea-derived *Micromonospora rosaria* SCSIO N160 [7]. In this study, we identified two novel phenylhydrazone derivatives, penzonemycins A–B (**1**–**2**), together with one new compound, dibenzoxazepinone demethylmycemycin A (**3**), and three known compounds (**4**–**6**) (Figure 1) from marine-derived *Streptomyces* sp. SCSIO 40020 [12]. Herein, we report the isolation and structural elucidation of penzonemycins A–B (**1**–**2**) and demethylmycemycin A (**3**), as well as a biological evaluation and a possible biosynthetic pathway of penzonemycins A–B (**1**–**2**).

## 2. Results and Discussion

### 2.1. Structural Determination

**Figure 2:**
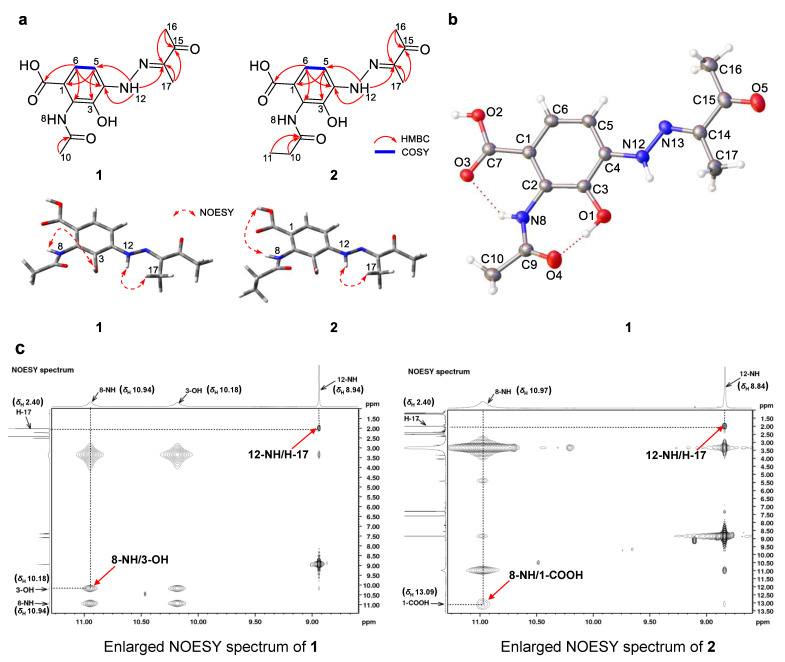
(**a**) Key HMBC, COSY and NOESY correlations for **1** and **2**; (**b**) X-ray crystallographic structure of **1**; (**c**) key NOESY correlations to support an hydroxyl group at C-3 and *E* configuration of the double bond Δ^13,14^ in **1** and **2**.

Penzonemycin A (**1**) was obtained as yellow needles. The molecular formula of **1** was determined to be C_13_H_15_N_3_O_5_ based on the HRMS (ESI-TOF) *m*/*z* [M + H]^+^, calcd for 294.1084, found 294.1086, indicating 8 degrees of unsaturation. The IR absorptions at 3360 and 1676 cm^−1^ suggested the presence of OH and C=O functionalities. The ^1^H NMR spectrum of **1** (Table 1) showed three singlet methyl groups, *δ*_H_ 2.21 (3H, s), 2.00 (3H, s) and 2.40 (3H, s), two aromatic *ortho* proton doublets *δ*_H_ 7.37 (1H, d, *J* = 8.6 Hz) and 7.57 (1H, d, *J* = 8.6 Hz), and four exchangeable proton signals *δ*_H_ 13.00 (1H, br s, 1-COOH), 10.94 (1H, br s, NH-8), 10.18 (1H, br s, 3-OH) and 8.94 (1H, br s, NH-12). The ^13^C NMR spectrum of **1** (Table 1, Figure S3) displayed 13 carbon signals for a 1,2,3,4-*tetra*-substituted benzene ring, three methyl groups, *δ*_C_ 23.6 (C-10), 8.1 (C-16) and 24.2 (C-17), and 4 quaternary carbons, *δ*_C_ 168.6 (C-7), 171.3 (C-9), 144.2 (C-13) and 196.3 (C-14). The COSY correlations of H-5 and H-6 and the HMBC correlations from H-5 to C-1/C-3 and from H-6 to C-2/C-4 confirmed the presence of a *tetra*-substituted benzene ring unit (Figure S3). The key HMBC correlations (Figure 2 and Figure S3) from H-6 to C-7 (*δ*_C_ 168.6) indicated a carboxylic acid group linked to the *tetra*-substituted benzene ring at C-1. The HMBC correlations from H_3_-10 (*δ*_H_ 2.21) to C-9 (*δ*_C_ 171.3) and the chemical shift value of C-9 revealed an acetamide group, which was deduced to be located at C-2 of the *tetra*-benzene ring by the chemical shift value of C-2 (*δ*_C_ 127.5). The HMBC correlations from H_3_-16 (*δ*_H_ 2.00)/H_3_-17 (*δ*_H_ 2.40) to C-14 (*δ*_C_ 144.2)/C-15 (*δ*_C_ 196.3) and from NH-12 to C-14, together with the remaining two N atoms, established a rare butanone hydrazone unit, which was linked to the benzene ring at C-4 by HMBC correlations from NH-12 to C-3/C-5. The remaining hydroxyl group (*δ*_H_ 10.18, 3-OH) was placed at C-3 based on the NOESY correlations of 3-OH and NH-8 (Figure 2). Thus, the planar structure of **1** was established. The *E* configuration of the double bond Δ^13,14^ in **1** was assigned by the NOESY correlation of NH-12 and H_3_-17 (Figure 2). Fortunately, high-quality crystals of **1** were obtained, allowing the successful performance of a single-crystal X-ray diffraction experiment using Cu K*a* radiation (CCDC 2131907), confirming the planar structure and absolute stereochemistry (*E*Δ^13,14^) of **1** (Figure 2, Table S4).

Penzonemycin B (**2**) was isolated as a yellowish solid. Its molecular formula was assigned as C_14_H_17_N_3_O_5_ by HRMS (ESI-TOF) *m*/*z* [M + H]^+^, calcd for 308.1241, found 308.1240, indicating 8 degrees of unsaturation. The IR spectrum of **2** displayed broad hydroxyl absorption at 3350 cm^−1^ and a carbonyl peak at 1676 cm^−1^. The ^1^H, ^13^C NMR data of **2** (Table 1, Figure S4) were highly similar to those of **1**. The difference was that **2** had a propionamide moiety instead of the acetylamino unit in **1**. The presence of the propionamide group in **2** was supported by the ^1^H-^1^H COSY correlation of H_2_-10 (*δ*_H_ 2.49) and H_3_-11 (*δ*_H_ 1.17) and HMBC correlations from H_3_-11 to C-9 (*δ*_C_ 174.5)/C-10 (Figure 2). The geometry of the double bond Δ^13,14^ in **2** was determined to be trans (*E*) by NOESY correlations of NH-12 and H_3_-17 (Figure 2).

**Figure 3:**
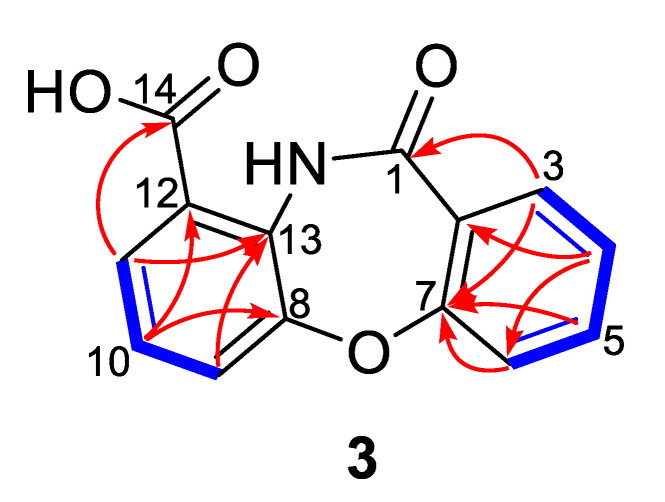
Key HMBC and COSY correlations for compound **3**.

Compound **3** was isolated as a white solid. The molecular formula of **3** was determined to be C_14_H_9_NO_4_ by HRMS (ESI-TOF) ([M + H]^+^, *m*/*z* 256.0616, calculated for 256.0604, Figure S5), indicating 11 degrees of unsaturation. The IR spectrum exhibited the presence of carbonyl (1716, 1633 cm^−1^) functional groups. The ^1^H NMR spectrum of **3** (Table 2, Figure S5) showed seven aromatic protons signals at *δ*_H_ 7.15 (1H, d, *J* = 8.3 Hz), 7.99 (1H, d, *J* = 8.6 Hz), 8.05 (1H, d, *J* = 7.8, 1.5 Hz), 8.12 (1H, d, *J* = 8.1 Hz), 7.11 (1H, d, *J* = 7.6 Hz), 7.57 (1H, d, *J* = 8.0, 1.7 Hz) and 7.58 (1H, d, *J* = 8.0 Hz). The ^13^C NMR spectrum of **3** (Table 2, Figure S5) displayed 14 carbon signals (Figure S5) for 7 methine carbons *δ*_C_ 115.4 (C-9), 117.4 (C-6), 120.1 (C-4), 125.4 (C-10), 127.4 (C-3), 127.5 (C-11) and 134.5 (C-5), 5 quaternary carbons signals *δ*_C_ 109.8 (C-2), 121.8 (C-12), 138.8 (C-13), 149.6 (C-8) and 158.4 (C-7) and 2 ester/amido carbonyl carbons signals *δ*_C_ 163.6 (C-1) and 165.6 (C-14). ^1^H and ^13^C NMR data of **3** (Table 2 and Figure S5) were highly similar to those of mycemycin A (**4**) [13]. Compound **3** was different from **4** by the absence of the methoxy group at C-14, which was supported by 14 mass units less compared to mycemycin A (**4**), as well as by a detailed analysis of 1D and 2D NMR spectra (Figure 3 and Figure S5). The COSY correlations of H-3 (*δ*_H_ 7.99)/H-4 (*δ*_H_ 7.11)/H-5 (*δ*_H_ 7.57)/H-6 (*δ*_H_ 7.15) and the HMBC correlations from H-4 to C-2/C-6, from H-3/H-5/H-6 to C-7, and from H-3 to C-1 established the salicylic acid fragment. The COSY correlations of H-9 (*δ*_H_ 8.12)/H-10 (*δ*_H_ 7.58)/H-11(*δ*_H_ 8.05) and the HMBC correlations from H-9/H-11 to C-13, from H-10 to C-12/C-8, and from H-11 to C-14 revealed a 3-hydroxyanthranilic acid unit. Given determined molecular formula as well as one less degree of unsaturation, two ring systems were connected by C-2-C-1-NH-C-13 and C-7-O-C-8. Thus, compound **3** was elucidated to be demethylmycemycin A.

**Figure 4:**
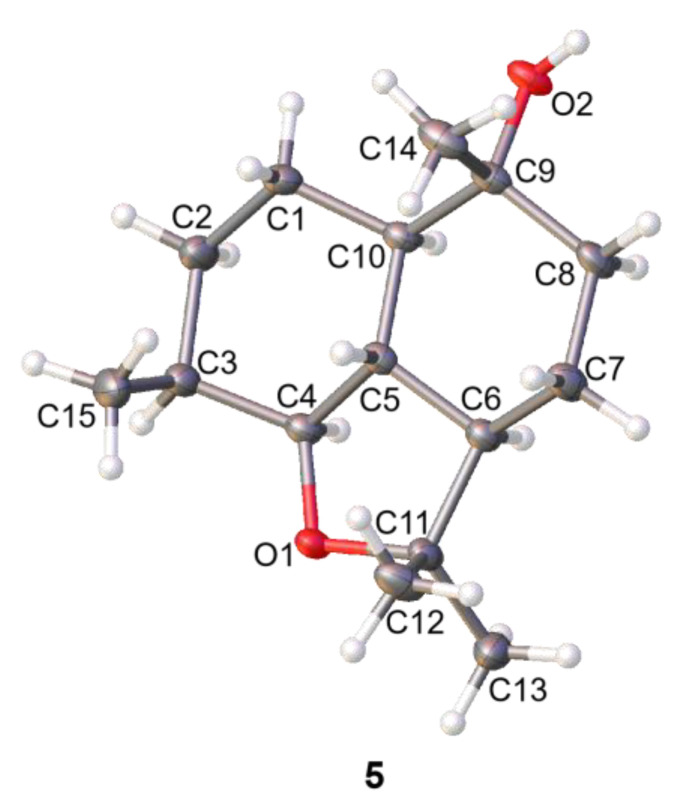
X-ray crystallographic structure of compound **5**.

Compounds **4**–**6** were identified as mycemycin A (**4**) [13], 9(*R*)-ganodermanol B (**5**) [14], and isopterocarpolone (**6**) [15], respectively. Their ^1^H and ^13^C NMR data (Figures S6–S8) were identical to those reported in the literature. In addition, we performed a crystallographic analysis of **5**. For the first time, the absolute configuration of **5** was confirmed by the way of single-crystal X-ray diffraction analysis (Figure 4) using Cu Kα data (CCDC 2177578) with Flack parameter of 0.04(9) (Table S5). 

### 2.2. Biosynthetic Implications

**Scheme 1:**
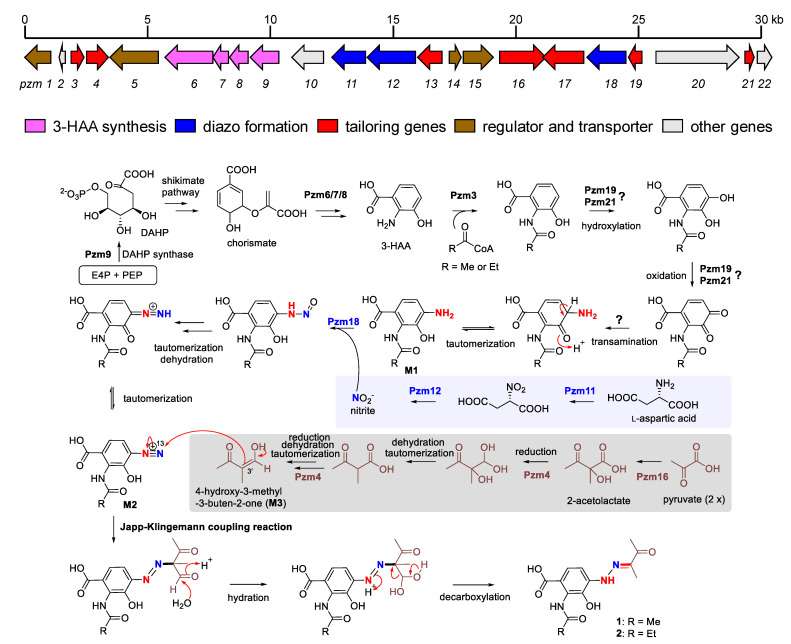
Plausible biosynthetic pathway of compounds **1**–**2**.

Penzonemycins A (**1**) and B (**2**) are new hydrazones harboring an −N−N=C− unit and a 3-hydroxyanthranilic acid (3-HAA) core. The whole genome of *Streptomyces* sp. SCSIO 40020 has been sequenced, revealing a putative gene cluster (*pzm*), spanning from *pzm1* to *pzm22* (Scheme 1, Table 3), that encodes the enzymatic machinery for the biosynthesis of **1** and **2**. The 3-HAA core was proposed to be derived from a chorismate pathway, given the presence of the genes *pzm9*, *pzm6*, *pzm7* and *pzm8*, which are predicted to encode 3-deoxy-D-arabino-heptulosonate-7-phosphate (DAHP) synthase, anthranilate synthase, isochorismatase and a dehydrogenase, respectively. Pzm9 catalyzes the condensation of D-erythrose-4-phosphate (E4P) and phosphoenolpyruvate (PEP) to form DAHP, the first intermediate in the shikimate pathway leading to chorismate [16,17]. The homologous proteins of Pzm6, Pzm7 and Pzm8 in the biosynthetic pathways of calcimycin and limazepine have been shown to be responsible for converting chorismate to 3-HAA (Scheme 1) [18,19]. Next, 3-HAA is putatively acylated by the N-acetyltransferase Pzm3 to afford an acetylated (or propionylated) product ready for two rounds of oxidations, likely catalyzed by the proposed oxidases Pzm19 and Pzm21. Subsequently, an unknown enzyme-catalyzed transamination occurs, followed by a tautomerization, to generate the putative intermediate **M1** (Scheme 1). The proposed oxidative transamination reactions are similar to the recent reported reactions in the formation of 8-amino-flaviolin during the biosynthesis of naphthoquinone-based meroterpenoid [20]. Notably, the three enzymes, namely, the nitrosuccinate lyase Pzm11, the monooxygenase Pzm12 and the AMP-binding protein Pzm18, are predicted to be the orthologues of CreD, CreE and CreM, respectively, which are responsible for the diazo formation in cremeomycin biosynthesis, utilizing L-aspartic acid as a nitrogen source [21]. Therefore, Pzm11 and Pzm12 are postulated to generate nitrite from L-aspartic acid, which is subsequently incorporated to **M1** by Pzm18, leading to the diazo group in **M2** after dehydration and tautomerization, similar to the biosynthesis of cremeomycin and triacsin [21,22]. Recently, an electrophilic diazo-anthranilate has been reported to undergo nonenzymatic Japp−Klingemann coupling with a *β*-keto aldehyde-containing precursor to furnish the hydrazone group in the biosynthesis of tasikamides [4]. Interestingly, Pzm16 encodes an acetolactate synthase and putatively catalyzes the coupling of two pyruvates to form 2-acetolactate [23], which becomes 4-hydroxy-3-methyl-3-buten-2-one (**M3**) after two rounds of carboxyl reduction (probably catalyzed by the F420-dependent oxidoreductase Pzm4) and spontaneous dehydration (Scheme 1). Finally, the nonenzymatic Japp−Klingemann coupling reaction between **M2** and **M3** constructs the hydrazone moiety in **1** and **2** after subsequent hydration and decarboxylation (Scheme 1).

### 2.3. Biological Activities

Penzonemycin A (**1**) was evaluated for antitumor activities in four human cancer cell lines SF-268, MCF-7, A549 and HepG-2 by the SRB method. Compound **1** showed weak activity in these three cancer cell lines (IC_50_ 30.44–61.92 µM) (Table S1). Penzonemycin A (**1**) was also assayed for its antibacterial activities, antifungal activities (Tables S2 and S3) and α-glucosidase inhibition activity. However, compound **1** showed no activities.

## 3. Materials and Methods

### 3.1. General Experimental Procedures

Optical rotations were measured using a 341 Polarimeter (Perkin-Elmer, Inc., Norwalk, CT, USA). UV spectra were measured on a U-2900 spectrophotometer (Hitachi, Tokyo, Japan). IR spectra were recorded on an Affinity-1 FT-IR spectrometer (Shimadzu, Tokyo, Japan). Melting points were measured using an SGW^®^X-5 micro melting point meter (Shanghai INESA Physico optiacal Instrument Co., Ltd., Shanghai, China). 1D and 2D NMR spectra were recorded on a Bruker AV-700 MHz NMR spectrometer (Bruker Biospin GmbH, Rheinstetten, Germany) with tetramethylsilane (TMS) as the internal standard. Mass spectrometric data were obtained on a quadrupole-time-of-flight mass spectrometer (Bruker Maxis 4G) for HRESIMS. Column chromatography was performed using silica gel (100–200 mesh, 300–400 mesh; Jiangyou Silica gel development, Inc., Yantai, China), Sephadex LH-20 (GE Healthcare Bio-Sciences AB, Uppsala, Sweden). HPLC was carried out while a reversed-phase column (Phenomenex Gemini C18, 250 mm × 4.6 mm, 5 μm; Phenomenex, Torrance, CA, USA) with UV detection at 254 nm and 360 nm. Semi-preparative HPLC was performed on a Hitachi HPLC station (Hitachi-L2130) with a Diode Array Detector (Hitachi L-2455) using a Phenomenex ODS column (250 mm × 10.0 mm, 5 μm; Phenomenex, Torrance, CA, USA) with UV detection at 360 nm.

### 3.2. Strain, Screening and Culture Methods

*Streptomyces* sp. SCSIO 40020 (original number LW701) was isolated from a marine sediment sample (E 114.0432°, N 22.0194°, Pearl River Estuary in China, at the depth of 28 m), and was identified by 16S rDNA sequence analysis (GenBank accession no. MW582618). The strain SCSIO 40020 was maintained in a 40% glycerol aqueous solution at −80 °C at the Research Center for Marine Microbiology Culture Collection Center of South China Sea Institute of Oceanology, Chinese Academy of Sciences. It was found that the strain SCSIO 40020 was best maintained on 38^#^-agar medium containing 3% sea salt for optimal growth and sporulation. A single colony was inoculated into 50 mL of four different media, including modifed-A1BFe+C [24] (soluble starch 1.0%, yeast extract 0.4%, tryptone 0.2%, CaCO_3_ 0.2%, sea salts 3%, pH 7.2–7.4), ZM7 medium (glycerol 0.5%, arginine 0.1%, glucose 0.1%, K_2_HPO_4_ 0.03%, MgSO_4_·7H_2_O 0.02%, NaCl 0.03%, vitamin B complex 0.5 mL, pH 7.2–7.4), AM6-4 [25] (glycerol 0.1%, bacterial peptone 0.5%, glycine 0.01%, alanine 0.01%, CaCO_3_ 0.5%, sea salts 3%, pH 7.2–7.4) and modifed-ISP3 [25] (oat meal 1.5%, FeSO_4_ 0.0001%, MnCl_2_ 0.0001%, ZnSO_4_ 0.0001%, sea salts 3%, pH 7.2–7.4), in 250 mL Erlenmeyer flasks and then incubated on a rotary shaker (200 rpm) at 28 °C for seven days. The culture broths were extracted with an equal volume of *n*-butanone, and the extracts were then monitored by HPLC-DAD. HPLC analyses were carried out using the following program: solvent system (solvent A, 10% acetonitrile in water supplemented with 0.08% formic acid; solvent B, 90% acetonitrile in water); 5% B to 100% B (linear gradient, 0–18 min), 100% B (18–23 min), 100% B to 5% B (23–27 min), 5% B (27–32 min); flow rate of 1 mL/min. A single colony was inoculated into 30 mL of modified A1BFe+C medium and incubated at 28 °C for 2–3 days. Then, 20 L of fermentation culture was prepared by inoculating 30 mL of the seed culture into a 1000 mL Erlenmeyer flask containing 200 mL of the modified ISP3 medium followed by cultivation on a rotary shaker (200 rpm) at 28 °C for 7 days.

### 3.3. Extraction, Isolation and Purification

The 20 L of culture broth of *Streptomyces* sp. SCSIO 40020 was centrifuged at 3900 rpm for 15 min at 25 °C. The mycelia were extracted three times, each with 2 L of acetone. The acetone extracts were concentrated under reduced pressure to afford an aqueous residue, which was extracted four times with an equal volume of *n*-butanone. The supernatants were extracted four times with an equal volume of *n*-butanone [12,24]. The butanone extracts were combined and concentrated under reduced pressure to afford the crude extracts (7.5 g). The crude extracts were subjected to column chromatography over silica gel, eluting with a gradient of CHCl_3_/MeOH mixtures i.e., 100/0, 95/5, 90/10, 80/20, 50/50 and 0/100 (*v*/*v*). We obtained five fractions (Fr.1–Fr.5). Then Fr.2 (3.50 g) was further purified via MPLC (Medium-Pressure Preparative Liquid Chromatography) with a reverse-phased C-18 column (14.5 cm × 2.5 cm i.d., 40–60 μm Agela Technologies, Torrance, CA, USA) by eluting with a linear gradient of H_2_O/MeOH (0–100%, 15 mL/min, 300 min), obtaining fractions Fr.2.1–Fr.2.10. Fractions Fr.2.5–Fr.2.6 (20 mg) were further purified by semi-preparative HPLC using a mobile phase of MeCN/H_2_O (50:50, *v*/*v*), which yielded compound **3** (1.8 mg) and compound **4** (1.5 mg). Fractions Fr.2.7–Fr.2.8 (200 mg) were further purified by semi-preparative HPLC using a mobile phase of MeCN/H_2_O (60:40, *v*/*v*), obtaining compound **1** (10.4 mg), compound **2** (2.2 mg), compound **5** (1.2 mg), and compound **6** (1.0 mg). 

### 3.4. Physical and Chemical Properties of the New Compounds ***1***–***3*** and ***5***

Penzonemycin A (**1**): yellow needles; m.p. 205.8–209.3 °C; [α${]}_{D}^{25}$: −1.8 (*c* 0.11, MeCN); UV (MeCN) λ_max_ (log ε): 358 (4.69) nm, 249 (4.50) nm, 210 (4.59) nm; IR ν_max_ 3360, 2947, 2837, 2359, 2332, 1676 cm^−1^, ^1^H NMR (700 MHz, DMSO-*d*_6_) and ^13^C NMR (176 MHz, DMSO-*d*_6_), see Table 1; (+)-HRESIMS *m*/*z* [M + H]^+^ 294.1086 (calculated for C_13_H_16_N_3_O_5_, 294.1084), [M + Na]^+^ 316.0908 (calculated for C_13_H_15_N_3_NaO_5_, 316.0904). 

Crystal data for penzonemycin A (**1**): C_13_H_15_N_3_O_5_ (*M* = 293.28 g/mol): monoclinic, space group P2_1_/n (no. 14), *a* = 4.8385 (2) Å, *b* = 19.6128 (7) Å, *c* = 14.5170 (6) Å, *β* = 98.487 (4), *V* = 1362.53 (9) Å^3^, *Z* = 4, *T* = 103 (5) K, μ (Cu Kα) = 0.945 mm^−1^, *Dcalc* = 1.430 g/cm^3^, 6318 reflections measured (9.018° ≤ 2Θ ≤ 148.158°), 2631 unique (*R*_int_ = 0.0376, R_sigma_ = 0.0368) which were used in all calculations. The final *R*_1_ was 0.0576 (I > 2σ(I)), and *wR*_2_ was 0.1809 (all data).

Penzonemycin B (**2**): a yellowish solid; [α${]}_{D}^{25}$: −1.1 (*c* 0.19, MeCN); UV (MeCN) λ_max_ (log ε): 358 (4.66) nm, 249 (4.48) nm, 210 (4.56) nm; IR ν_max_ 3350, 2358, 1676, 1267, 667 cm^−1^; ^1^H and ^13^C NMR data, see Table 1; (+)-HRESIMS *m*/*z* [M + H]^+^ 308.1240 (calculated for C_14_H_18_N_3_O_5_, 308.1241), [M + Na]^+^ 330.1054 (calculated for C_13_H_15_N_3_NaO_5_, 330.1060). 

Compound **3**: a white solid; [α${]}_{D}^{25}$: +8.9 (*c* 0.20, MeCN); UV (CHCl_3_) λ_max_ (log ε): 346 (4.07) nm, 335 (4.09) nm; 306 (3.92) nm; 264 (3.94) nm, 247 (3.89) nm; IR ν_max_ 2953, 2914, 1716, 1633, 1548, 1489, 1303, 1259, 1244, 1192, 1159, 1060, 875, 786, 748, 717, 686 cm^−1^, ^1^H and ^13^C NMR data, see Table 2; (+)-HRESIMS *m*/*z* [M + H]^+^ 256.0616 (calculated for C_14_H_18_N_3_O_5_, 256.0604). 

Crystal data for 9(*R*)-ganodermanol B (**5**): C_15_H_26_O_2_ (*M* = 238.36 g/mol): orthorhombic, space group P2_1_/n (no. 19), *a* = 8.35172 (11) Å, *b* = 11.17809 (15) Å, *c* = 14.82535 (19) Å, *V* = 1384.04 (3) Å^3^, *Z* = 4, *T* = 99.8 (8) K, μ (Cu Kα) = 0.570 mm^−1^, *Dcalc* = 1.144 g/cm^3^, 7345 reflections measured (9.91° ≤ 2Θ ≤ 148.416°), 2727 unique (*R*_int_ = 0.0278, R_sigma_ = 0.0319) which were used in all calculations. The final *R*_1_ was 0.0355 (I > 2σ(I)), and *wR*_2_ was 0.0946 (all data).

### 3.5. X-Ray Crystallographic Analysis Data of Penzonemycin A (***1***) and ***5***

Single crystals of C_13_H_15_N_3_O_5_ (penzonemycin A **1**) and C_15_H_26_O_2_ (9(*R*)-ganodermanol B **5**) were obtained in MeOH solution. Their data were collected on an XtaLAB AFC12 (RINC): Kappa single diffractometer. The crystals were kept at 100.00 (10) K during data collection. Using Olex2 [26], their structures were solved with the ShelXT [27] structure solution program using Intrinsic Phasing and refined with the ShelXL [28] refinement package, using Least Squares minimization. Crystallographic data for **1** and **5** were deposited at the Cambridge Crystallographic Data Center (CCDC) with the deposition numbers 2,131,907 and 2,177,578, respectively.

### 3.6. Bioactivity Assays

#### 3.6.1. Cytotoxic Activity Assay

The in vitro cytotoxic activities of penzonemycin A (**1**) were evaluated in four tumor cell lines, i.e., SF-268 (human glioma cell line), HepG-2 (human liver carcinoma cell line), MCF-7 (human breast adenocarcinoma cell line), and A549 (human lung adenocarcinoma cell) by the SRB method [24], according to a previously described protocol [29]. The cells were cultivated in RPMI 1640 medium [30]. Cells (180 μL) with a density of 3 × 10^4^ cells/mL were seeded onto 96-well plates and incubated for 24 h at 37 °C, 5% CO_2_. Subsequently, 20 μL of different concentrations of penzonemycin A (**1**), ranging from 0 to 100 µM in dimethyl sulfoxide (DMSO), was added to each plate well. An equal volume of DMSO was used as a negative control. After further incubation for 72 h, the cell monolayers were fixed with 50% (*wt*/*v*) trichloroacetic acid (50 μL) and then stained for 30 min with 0.4% (*wt*/*v*) SRB dissolved in 1% acetic acid. The unbound dye was removed by repeatedly washing with 1% acetic acid. The protein-bound dye was dissolved in 10 mM Tris-base solution (200 µL) for the determination of the optical density (OD) at 570 nm using a microplate reader. The cytotoxic compound cisplatin was used as a positive control. All data were obtained in triplicate and are presented as means ± S.D. IC_50_ values were calculated with the SigmaPlot 14.0 software using the non-linear curve-fitting method.

#### 3.6.2. Antifungal Activity Assay

The antifungal activity of compound **1** was measured against three phytopathogenic fungi, i.e., *Colletotrichum gloeosporioides* Penz, *Physalospora piricola* Nose, *Bipolaris sorokiniana*, by the broth microdilution method [31]. Compound **1** was dissolved in DMSO at the final concentration of 2.56 mg mL^−1^. The indicator fungi strains were grown for 48 h on a rotary shaker at 28 °C. The cultures were diluted with sterilized medium to achieve a predetermined optical absorbance at 600 nm, then diluted 1000-fold before being added into 96-well microtiter plates. Three replicates of each compound were tested in dilution series, ranging from 128 to 0.25 μg mL^−1^. The lowest concentrations that completely inhibited the visible growth of the tested strains were recorded after 48 h of cultivation from two independent experiments. Nystatin was used as a positive control against the three phytopathogenic fungi.

#### 3.6.3. Antibacterial Activity Assay

The antibacterial activity of compound **1** was assayed against *Escherichia coli* ATCC 25922, *Acinetobacter baumannii* ATCC 19606, *Staphyloccocus aureus* ATCC 29213, and Methicillin-resistant *S. aureus* (MRSA) ATCC 43300 by the broth microdilution method [31]. Compound **1** was dissolved in DMSO at the final concentration of 2.56 mg mL^−1^. The indicator strain was grown for 24 h on a rotary shaker at 37 °C. The cultures were diluted with sterilized medium to achieve a predetermined optical absorbance at 600 nm, then diluted 1000-fold before being placed into 96-well microtiter plates. Three replicates of each compound were tested in dilution series, ranging from 128 to 0.25 μg mL^−1^. The MIC values were recorded after 24 h of cultivation from two independent experiments. Ciprofloxacin and vancomycin were used as positive controls.

#### 3.6.4. Alpha-glucosidase Inhibition Activity

A general inhibiting reaction assay contained 0.04 U/mL, 2.0 mM *p*-nitrophenyl-*α*-D-glucopyranoside solution (Shanghai Macklin Biochemical Co., Ltd., Shanghai, China) and a specific concentration of **1** and was carried out in PBS buffer (50 mM, pH 7.0), in a total volume of 100 μL. In detail, first, sample **1** was mixed with *α*-glucosidase and incubated at 37 °C for 10 min, and then, the reactions were initiated by adding the *p*-nitrophenyl-*α*-D-glucopyranoside solution and continued at 37 °C for another 15 min. After that, the absorbance at 405 nm was recorded on a microplate reader (Enspire, PerkinElmer, Singapore) within 30 min. In the assay, DMSO was used as a negative control, and acarbose (Shanghai Aladdin Biochemical Technology Co., Ltd., Shanghai, China) was used as a positive control. All treatments were performed in triplicate, and the IC_50_ value was determined by regression analysis [32].

### 3.7. Bioinformatics Analysis

The genomic DNA of *Streptomyces* sp. SCSIO 40020 was isolated according to a prior study [12]. The whole genome was sequenced and assembled by Nextomics Biosciences Co., Ltd. (Wuhan, China) using Oxford Nanopore GridION and canu. The putative biosynthetic gene clusters in the genome were predicted with antiSMASH 6.0 [33]. The deduced ORFs were analyzed using online 2ndFind software (https://biosyn.nih.go.jp/2ndfind/, accessed on 13 January 2022), and their functional predictions were accomplished with an online BLAST program (https://blast.ncbi.nlm.nih.gov/Blast.cgi, accessed on 13 January 2022). The DNA sequences of the penzonemycins gene cluster were deposited in GenBank, accession number ON345781. 

## 4. Conclusions

In conclusion, two new phenylhydrazone derivatives and a new alkaloid, penzonemycins A–B (**1**–**2**) and demethylmycemycin A (**3**), together with three known compounds (**4**–**6**) were obtained from the Pearl River Estuary sediment-derived *Streptomyces* sp. SCSIO 40020. Penzonemycins feature a hydrazone unit (−N−N=C−) that is rare in natural products. Biosynthetically, a Japp−Klingemann reaction is proposed as a key reaction leading to the hydrazone moiety in **1** and **2**, which is worth of further investigations.

## Figures and Tables

**Table 1:** ^1^H (700 MHz) and ^13^C NMR (176 MHz) data for **1** and **2** in DMSO-*d*_6_.

Position	1		2	
	*δ*_C_, Type	*δ*_H_, Mult (*J* Hz)	*δ*_C_, Type	*δ*_H_, Mult (*J* Hz)
1	115.3, C		117.3, C	
2	127.5, C		128.0, C	
3	137.6, C		136.1, C	
4	137.0, C		136.4, C	
5	109.4, CH	7.37, d (8.6)	108.6, CH	7.30, d (8.5)
6	124.0, CH	7.57, d (8.6)	123.6, CH	7.58, d (8.5)
7	168.6, C		169.2, C	
8-NH		10.94, br s		10.97, br s
9	171.3, C		174.5, C	
10	23.6, CH_3_	2.21, s	29.8, CH_2_	2.49, overlapping
11			9.8, CH_3_	1.17, t (7.6)
12-NH		8.94, s		8.84, s
14	144.2, C		143.3, C	
15	196.3, C		196.2, C	
16	8.1, CH_3_	2.00, s	7.9, CH_3_	1.99, s
17	24.2, CH_3_	2.40, s	24.1, CH_3_	2.40, s
3-OH		10.18, br s		n.d.
1-COOH		13.00, br s		13.09, br s

“n.d.”: not detection. Data were recorded on a Bruker Avance 700 MHz NMR spectrometer in DMSO-*d*_6_ with TMS as an internal standard. The signals were assigned with the aid of ^1^H-^1^H COSY, HSQC, and HMBC data.

**Table 2:** ^1^H (700 MHz) and ^13^C NMR (176 MHz) Data for **3** in DMSO-*d*_6_.

Position	3	
	*δ*_C_, Type	*δ*_H_, Mult (*J* in Hz)
1	163.6, C	
2	109.8, C	
3	127.4, CH	7.99, dd (7.7, 0.8)
4	120.1, CH	7.11, t (7.6)
5	134.5, CH	7.57, t (8.0, 1.7)
6	117.4, CH	7.15, d (8.3)
7	158.4, C	
8	149.6, C	
9	115.4, CH	8.12, d (8.1)
10	125.4, CH	7.58, t (8.0)
11	127.5, CH	8.05, dd (7.8, 1.5)
12	121.8, C	
13	138.8, C	
14	165.6, C	

Data were recorded on a Bruker Avance 700 MHz NMR spectrometer in DMSO-*d*_6_, with TMS as an internal standard. The signals were assigned with the aid of ^1^H-^1^H COSY, HSQC, and HMBC data.

**Table 3:** List of genes in the **p**en**z**one**m**ycin (*pzm*) biosynthetic gene cluster and their proposed functions. The DNA sequence of the *pzm* gene cluster has been deposited in GenBank (accession: ON345781).

Gene	Size (aa)	Annotation and Closest Homologue (Source, Accession No.)	% Identity/ Similarity
*pzm1*	353	LysR family transcriptional regulator (*Streptomyces halstedii* LGO-A4, MBV7672918)	94/94
*pzm2*	83	hypothetical protein (*Streptomyces halstedii* LGO-A4, MBV7672919)	100/100
*pzm3*	171	*N*-acetyltransferase (*Streptomyces halstedii* LGO-A4, MBV7672920)	98/98
*pzm4*	298	F420-dependent oxidoreductase (*Streptomyces halstedii* LGO-A4, MBV7672921)	99/99
*pzm5*	653	AfsR/SARP family transcriptional regulator (*Streptomyces* sp. SM18, AWL43113)	99/99
*pzm6*	650	anthranilate synthase (*Streptomyces* *halstedii* LGO-A4, MBV7672922)	99/99
*pzm7*	207	isochorismatase (*Streptomyces halstedii* LGO-A4, MBV7672923)	99/100
*pzm8*	256	2,3-dihydro-2,3-dihydroxybenzoate dehydrogenase (*Streptomyces halstedii* LGO-A4, MBV7672924)	98/99
*pzm9*	393	3-deoxy-d-arabino-heptulosonate 7-phosphate (DAHP) synthase(*Streptomyces halstedii* LGO-A4, MBV7672925)	99/98
*pzm10*	432	transposase (*Streptomyces halstedii* LGO-A4, MBV7672926)	99/99
*pzm11*	459	nitrosuccinate lyase, CreD homolog(*Streptomyces cremeus* NBRC 12760, BAU09301)	67/73
*pzm12*	659	monooxygenase, CreE homolog (*Streptomyces cremeus* NBRC 12760, BAU09302)	62/70
*pzm13*	334	quinone reductase (*Streptomyces halstedii* LGO-A4, MBV7672929)	99/99
*pzm14*	161	Rrf2 family transcriptional regulator (*Streptomyces halstedii* LGO-A4, MBV7672930)	100/100
*pzm15*	406	major facilitator superfamily (MFS) transporter (*Streptomyces halstedii* LGO-A4, MBV7672931)	100/100
*pzm16*	622	acetolactate synthase (*Streptomyces halstedii* LGO-A4, MBV7672932)	99/99
*pzm17*	560	Acyl-CoA ligase (*Streptomyces halstedii* LGO-A4, MBV7672933)	99/99
*pzm18*	527	AMP-binding protein, CreM homolog (*Streptomyces cremeus* NBRC 12760, BAU09310)	48/61
*pzm19*	180	SRPBCC family protein (*Streptomyces halstedii* LGO-A4, MBV7672935)	99/99
*pzm20*	1128	discoidin domain-containing protein (*Streptomyces halstedii* LGO-A4, MBV7672936)	99/99
*pzm21*	126	vicinal oxygen chelate (VOC) family protein (*Streptomyces halstedii* LGO-A4, MBV7672937)	100/100
*pzm22*	198	RNA 2′,3′-cyclic phosphodiesterase (*Streptomyces halstedii* LGO-A4, MBV7672938)	99/100

## Data Availability

The data presented in this study are available on request from the corresponding author.

## Supplementary Materials

The following supporting information can be downloaded at: https://www.mdpi.com/article/10.3390/md20070449/s1, Table S1: Cytotoxicity of penzonemycin A (**1**). Table S2: Antifungal activity of penzonemycin A (**1**). Table S3: Antibacterial activity of penzonemycin A (**1**). Table S4: Crystal data and structure refinement for penzonemycin A (**1**). Table S5: Crystal data and structure refinement for 9(*R*)-ganodermanol B (**5**). Figure S1: Natural hydrazone-containing compounds reported in the literature. Figure S2: HPLC analysis of the metabolic profiles of *Streptomyces* sp. SCSIO 40020 cultured in different media. Figure S3: Spectral data for penzonemycin A (**1**). Figure S4: Spectral data for penzonemycin B (**2**). Figure S5: Spectral data for demethylmycemycins A (**3**). Figure S6: Spectral data for mycemycin A (**4**). Figure S7: Spectral data for 9(*R*)-ganodermanol B (**5**). Figure S8: Spectral data for isopterocarpolone (**6**).
