# Supplementary material for: Two New Phenylhydrazone Derivatives from the Pearl River Estuary Sediment-Derived Streptomyces sp. SCSIO 40020
Source: Mar Drugs. 2022 Jul 9;20(7):449. doi: 10.3390/md20070449 (PMC9323291; doi:10.3390/md20070449)

Supplementary Materials

Two New Phenylhydrazone Derivatives from the Pearl River Estuary sediment-derived *Streptomyces* sp. SCSIO 40020

Wei Liu[1,2], Liang Ma[1,3], Liping Zhang[1,3], Yuchan Chen[2], Qingbo Zhang[1,3], Haibo Zhang[1,3], Weimin Zhang[2], Changsheng Zhang[1,3,4]*, Wenjun Zhang[1,3,4]*

[1] Key Laboratory of Tropical Marine Bio-resources and Ecology, Guangdong Key Laboratory of Marine Materia Medica, South China Sea Institute of Oceanology, Innovation Academy for South China Sea Ecology and Environmental Engineering, Chinese Academy of Sciences, 164 West Xingang Road, Guangzhou 510301, China; wwl1430@outlook.com (W.L.); maliangyc@hotmail.com (L.M.); zhanglp@scsio.ac.cn (L.Z.); gudaobo@163.com (Q.Z.); zhanghb@scsio.ac.cn (H.Z.)

[2] State Key Laboratory of Applied Microbiology Southern China, Institute of Microbiology, Guangdong Academy of Sciences, 100 Central Xianlie Road, Guangzhou 510070, China; chenyc@gdim.cn (Y.C.); wmzhang@gdim.cn (W.Z.)

[3] Southern Marine Science and Engineering Guangdong Laboratory (Guangzhou), No.1119, Haibin Road, Guangzhou 511458, China

[4] University of Chinese Academy of Sciences, Beijing 100049, China

* Correspondence: czhang@scsio.ac.cn (C.Z.); wzhang@scsio.ac.cn (W.Z.) Tel.: +86-20-8902-3038

Content

Table S1. Cytotoxicities of penzonemycin A (**1**). S3

Table S2. The antifungal activities of penzonemycin A (**1**)...S3

Table S3. The antibacterial activities of penzonemycin A (**1**). S3

Table S4. Crystal data and structure refinement for penzonemycin A (**1**)...........................S4

Table S5. Crystal data and structure refinement for 9(*R*)-ganodermanol B (**5**)....................S5

Figure S1. Natural hydrazone-containing compounds reported in literatures......................S6

Figure S2. HPLC analysis of metabolite profiles of *Streptomyces* sp. SCSIO 40020 cultured in different media. S7

Figure S3. Spectral data for penzonemycin A (**1**). S8

Figure S4. Spectral data for penzonemycin B (**2**). S15

Figure S5. Spectral data for demethylmycemycin A (**3**). S22

Figure S6. Spectral data for mycemycin A (**4**). S28

Figure S7. Spectral data for 9(*R*)-ganodermanol B (**5**). S30

Figure S8. Spectral data for isopterocarpolone (**6**). S32

Table S1. Cytotoxicities of penzonemycin A (**1**).

	**IC_{50} (μM)**			
	**SF-268**	**MCF-7**	**A549**	**HepG-2**
**1**	37.76 ± 1.83	>100	63.19 ± 0.73	31.31 ± 0.87
[a] **CP**	3.26 ± 0.29	3.19 ± 0.12	1.56 ± 0.08	2.42 ± 0.14

[a] Cisplatin, positive control.

Table S2. The antifungal activities of penzonemycin A (**1**)

	MIC (μg/mL)		
	Colletotrichum gloeosporioides Penz	*Physalospora piricola* Nose	*Bipolaris sorokiniana*
**1**	>64	>64	>64
[a] nystatin	2.0	0.5	0.5

[a] nystatin, positive control.

Table S3. The antibacterial activities of penzonemycin A (**1**).

	MIC (μg/mL)			
	Escherichia coli ATCC 25922	*Acinetobacter baumannii* ATCC 19606	*Staphyloccocus aureus* ATCC 29213	MRSA ATCC 43300
**1**	>64	>64	>64	>64
**Control**	1.0[a]	1.0[a]	0.5[b]	0.5[b]

a. ciprofloxacin, b. vancomycin

Table S4. Crystal data and structure refinement for penzonemycin A (**1**).

Identification code	penzonemycin A (**1**)
Empirical formula	$C_{13}H_{15}N_3O_5$
Formula weight	293.28
Temperature/K	103(5)
Crystal system	monoclinic
Space group	$P2_1/n$
a/Å	4.8385(2)
b/Å	19.6128(7)
c/Å	14.5170(6)
α/°	90
β/°	98.487(4)
γ/°	90
Volume/Å^3	1362.53(9)
Z	4
ρ_{calc}g/cm^3	1.430
μ/mm^{-1}	0.945
F(000)	616.0
Crystal size/mm^3	0.1 × 0.02 × 0.02
Radiation	Cu Kα (λ = 1.54184)
2Θ range for data collection/°	9.018 to 148.158
Index ranges	$-5 \leq h \leq 3$, $-24 \leq k \leq 23$, $-16 \leq l \leq 18$
Reflections collected	6318
Independent reflections	2631 [R_{int} = 0.0376, R_{sigma} = 0.0368]
Data/restraints/parameters	2631/0/202
Goodness-of-fit on F^2	1.075
Final R indexes [I>=2σ (I)]	R_1 = 0.0576, wR_2 = 0.1756
Final R indexes [all data]	R_1 = 0.0649, wR_2 = 0.1809
Largest diff. peak/hole / e Å^{-3}	0.34/-0.34

Table S5. Crystal data and structure refinement for 9(*R*)-ganodermanol B (**5**)

Identification code	9(*R*)-ganodermanol B (**5**)
Empirical formula	$C_{15}H_{26}O_2$
Formula weight	238.36
Temperature/K	99.8(8)
Crystal system	orthorhombic
Space group	$P2_12_12_1$
a/Å	8.35172(11)
b/Å	11.17809(15)
c/Å	14.82535(19)
α/°	90
β/°	90
γ/°	90
Volume/Å^3	1384.04(3)
Z	4
ρ_{calc}g/cm^3	1.144
μ/mm^{-1}	0.570
F(000)	528.0
Crystal size/mm^3	0.1 × 0.05 × 0.05
Radiation	CuKα (λ = 1.54184)
2Θ range for data collection/°	9.91 to 148.416
Index ranges	$-8 \leq h \leq 10, -13 \leq k \leq 6, -17 \leq l \leq 17$
Reflections collected	7345
Independent reflections	2727 [R_{int} = 0.0278, R_{sigma} = 0.0319]
Data/restraints/parameters	2727/0/159
Goodness-of-fit on F^2	1.056
Final R indexes [I>=2σ (I)]	R_1 = 0.0355, wR_2 = 0.0934
Final R indexes [all data]	R_1 = 0.0369, wR_2 = 0.0946
Largest diff. peak/hole / e Å^{-3}	0.23/-0.18
Flack parameter	0.04(9)

Figure S1. Natural hydrazone-containing compounds reported in literatures.

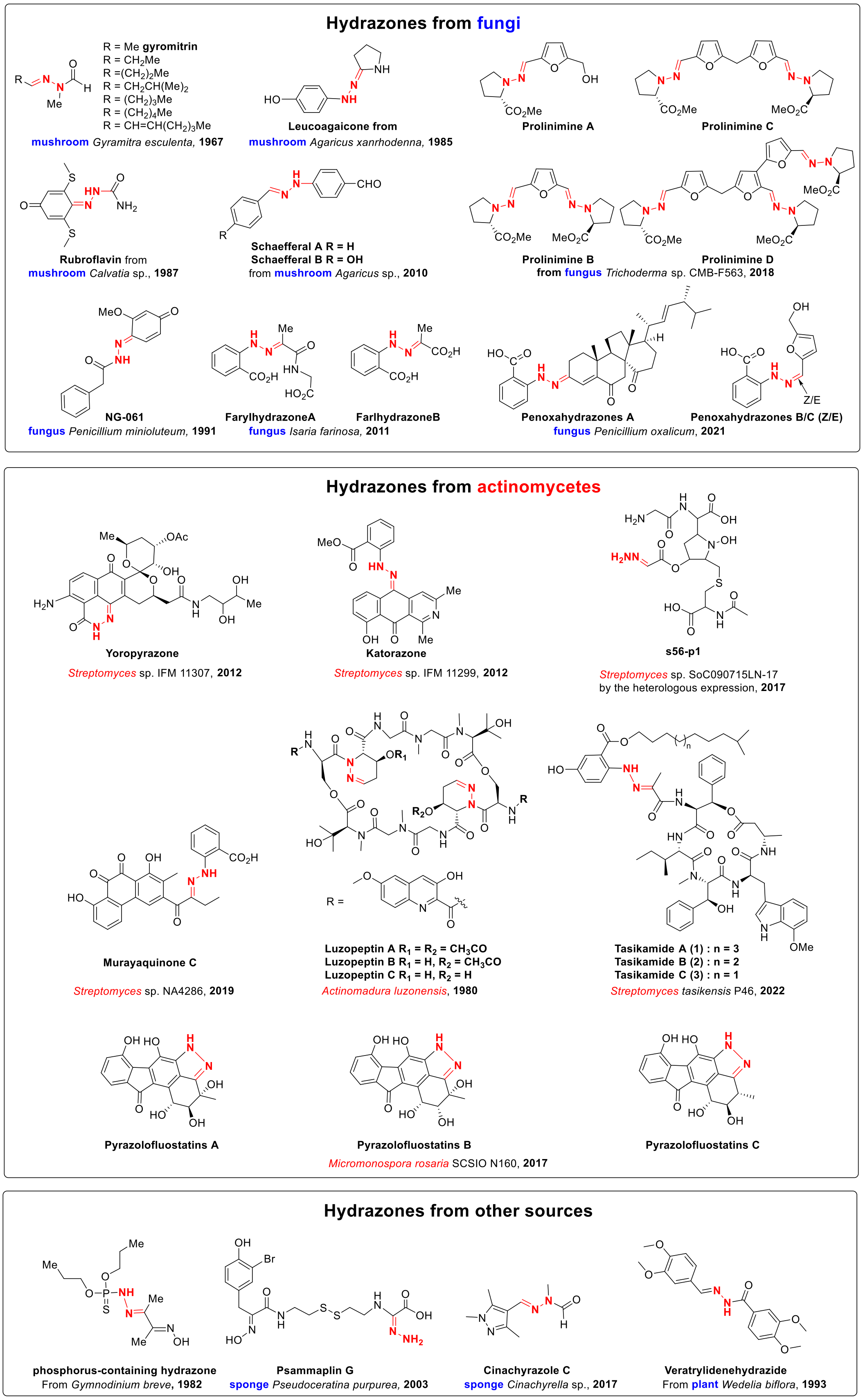

Figure S2. HPLC analysis of metabolite profiles of *Streptomyces* sp. SCSIO 40020 cultured in different media.

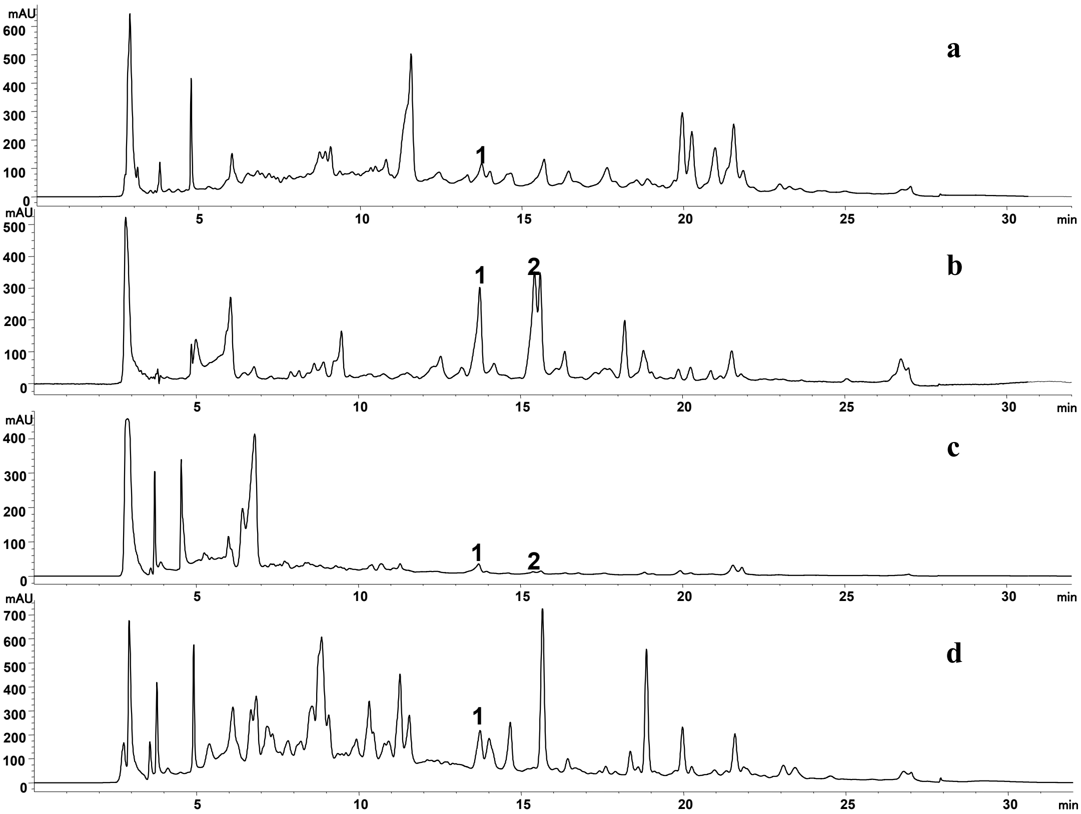

(**a**) modifed-A1BFe+C (soluble starch 1.0%, yeast extract 0.4%, tryptone 0.2%, $CaCO_3$ 0.2%, sea salts 3%, pH 7.2–7.4); (**b**) Modified-ISP3 (oatmeal 1.5%, $FeSO_4$ 0.0001%, $MnCl_2$ 0.0001%, $ZnSO_4$ 0.0001%, sea salts 3%, pH 7.2–7.4). (**c**) ZM7 medium (glycerol 0.5%, arginine 0.1%, glucose 0.1%, K_2HPO_4 0.03%, $MgSO_4 \cdot 7H_2O$ 0.02%, NaCl 0.03%, vitamin B complex 0.5ml, pH 7.2–7.4); (**d**) AM6-4 (glycerol 0.1%, bacterial peptone 0.5%, glycine 0.01%, alanine 0.01%, $CaCO_3$ 0.5%, sea salts 3.0%, pH 7.2-7.4).

Figure S3. Spectral data for penzonemycin A (**1**).

(A) HRESIMS

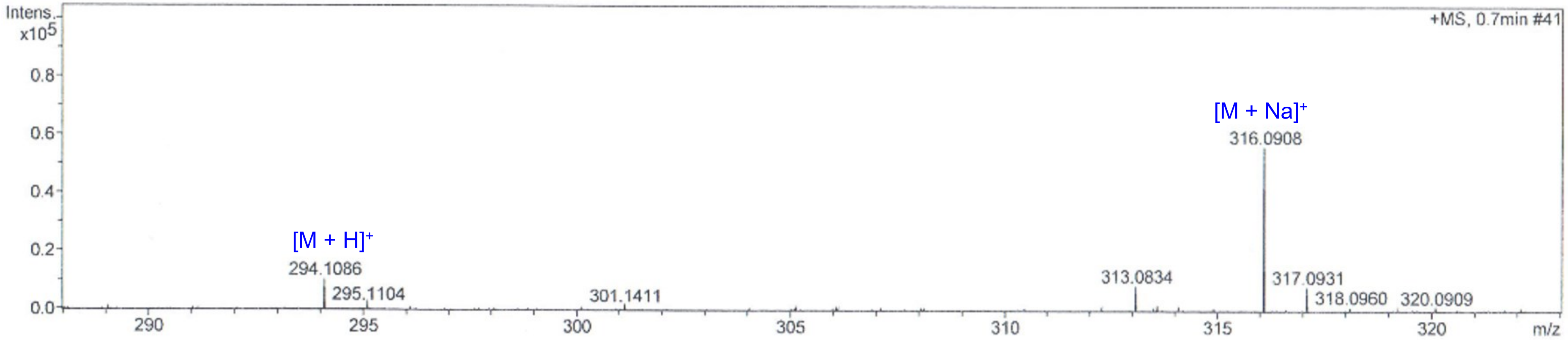

(**B**) IR

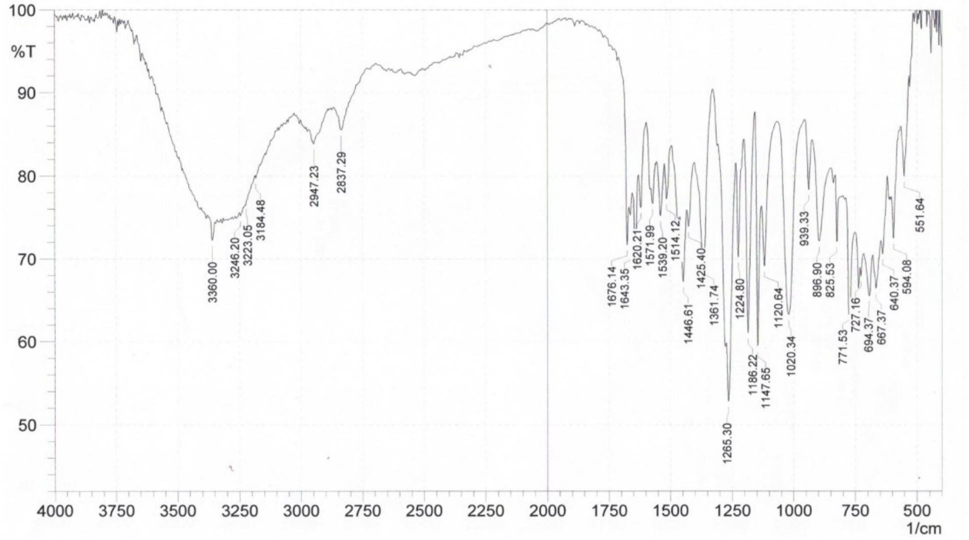

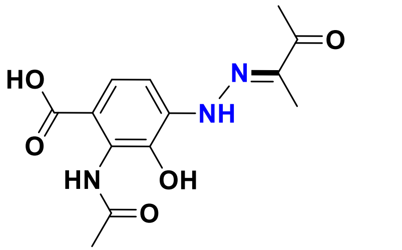

1

Chemical Formula: $C_{13}H_{15}N_3O_5$

caculated for $[M + H]^+$:294.1084

caculated for $[M + Na]^+$:316.0904

Figure S3. Spectral data for penzonemycin A (**1**) (continued).

(C) The ^{1}H NMR spectrum of penzonemycin A (**1**) in DMSO-d_6.

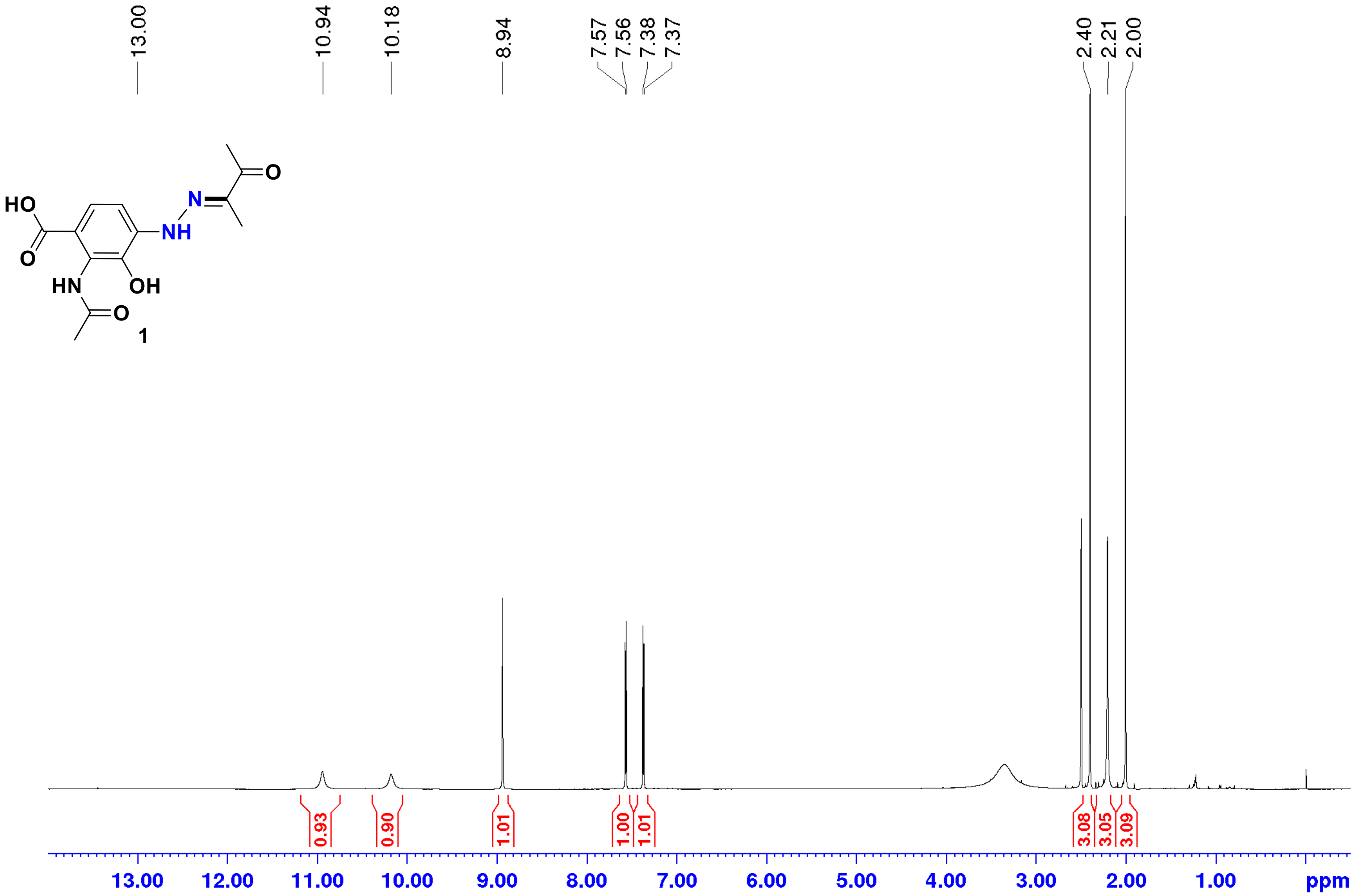

Figure S3. Spectral data for penzonemycin A (**1**) (continued).

(D) The ^{13}C NMR spectrum of penzonemycin A (**1**) in DMSO-d_6.

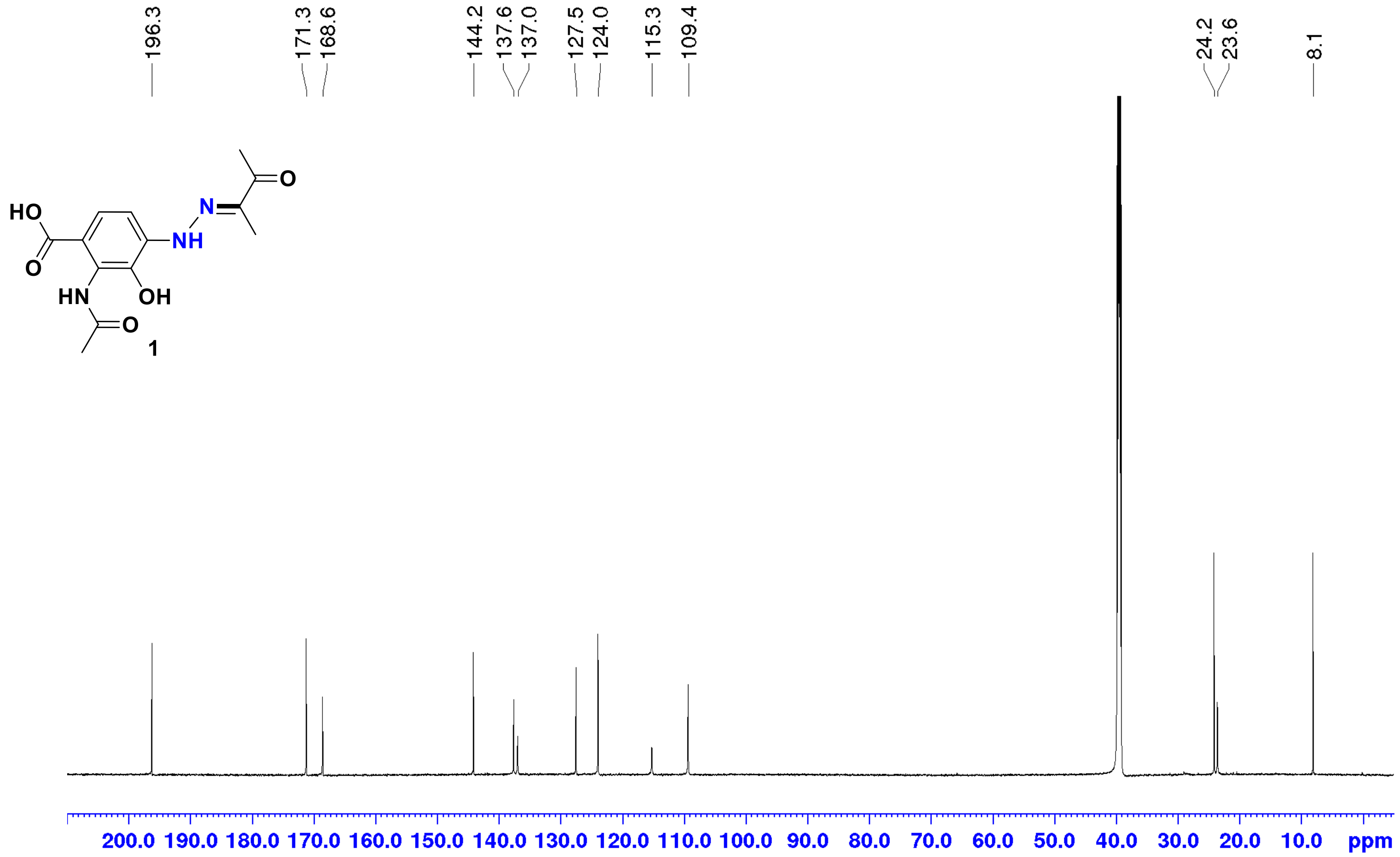

Figure S3. Spectral data for penzonemycin A (**1**) (continued).

(E) The HSQC spectrum of penzonemycin A (**1**) in DMSO-d_6.

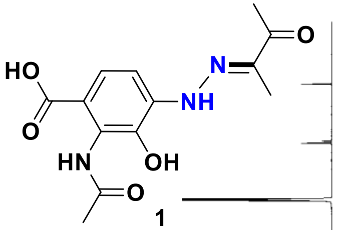

1

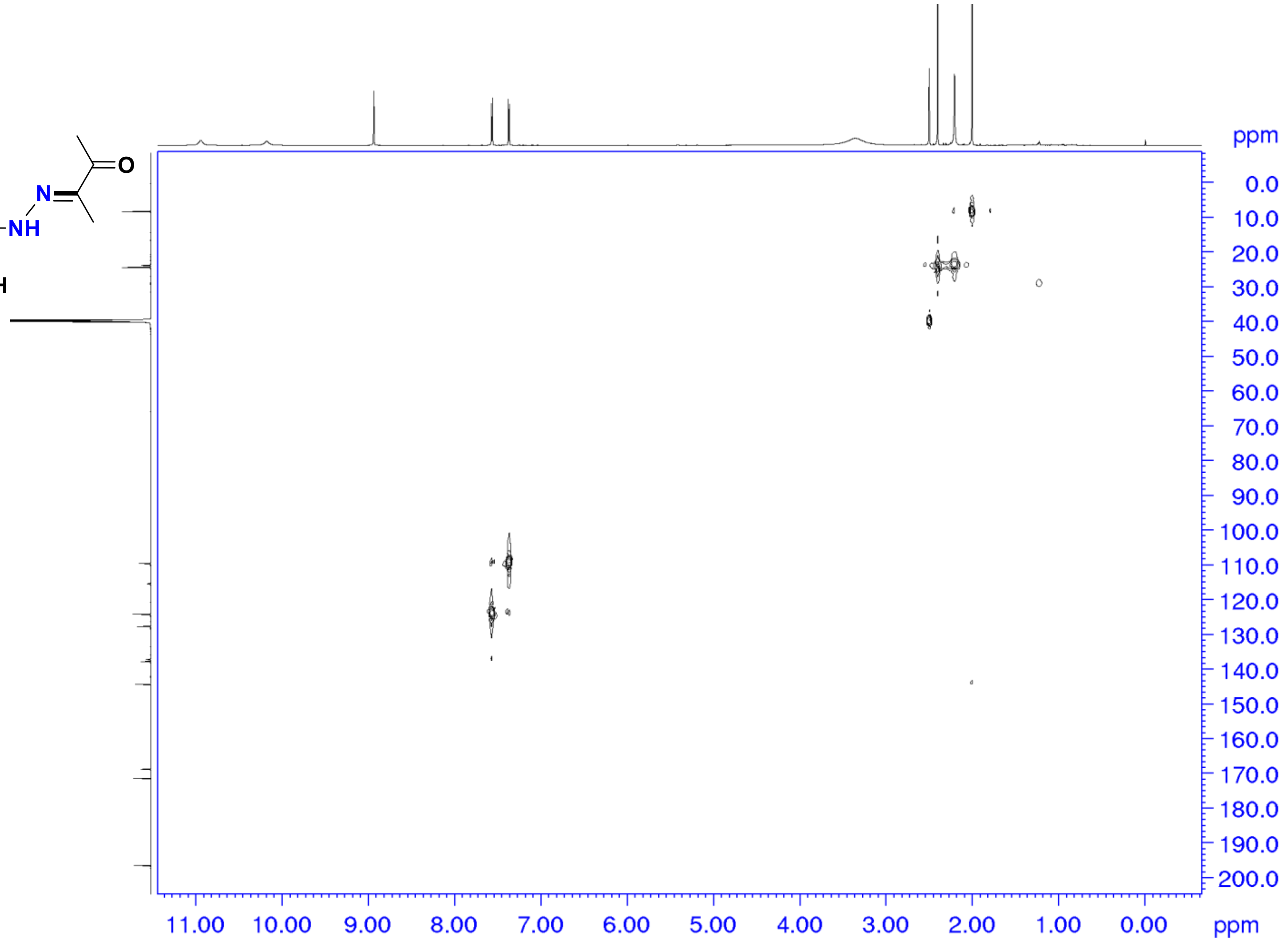

Figure S3. Spectral data for penzonemycin A (**1**) (continued).
(F) The HMBC spectrum of penzonemycin A (**1**) in DMSO-d_6.

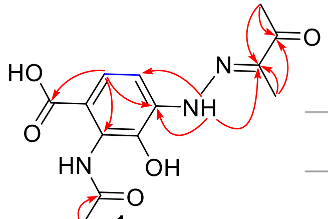

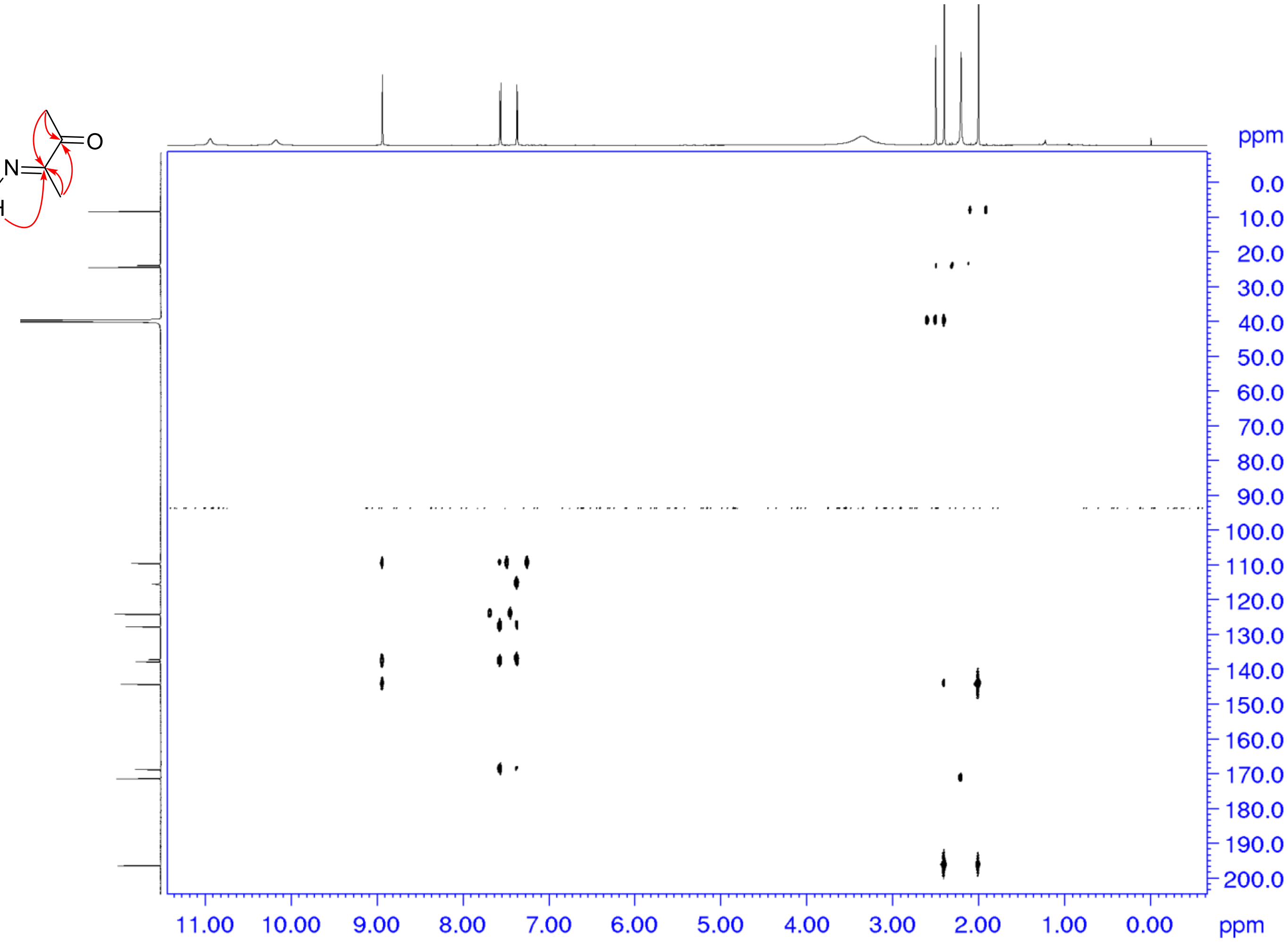

Figure S3. Spectral data for penzonemycin A (**1**) (continued).

(G) The ^{1}H-^{1}H COSY spectrum of penzonemycin A (**1**) in DMSO-d_6.

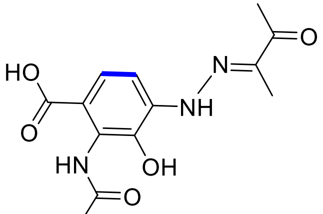

1

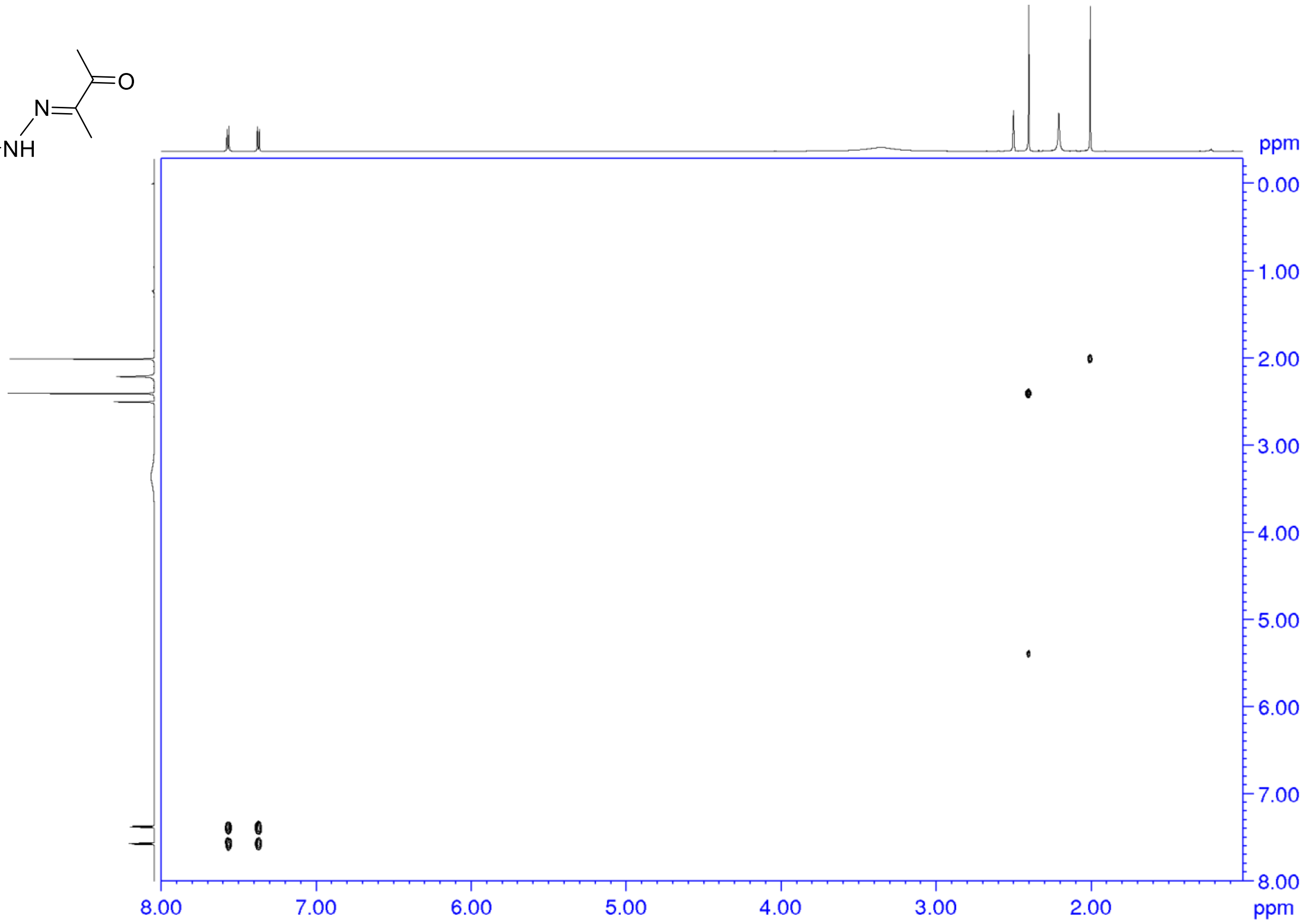

Figure S3. Spectral data for penzonemycin A (**1**) (continued).

(H) The NOESY spectrum of penzonemycin A (**1**) in DMSO-d_6.

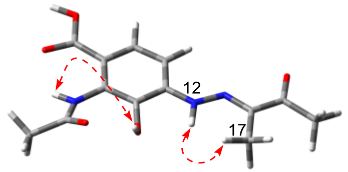

1

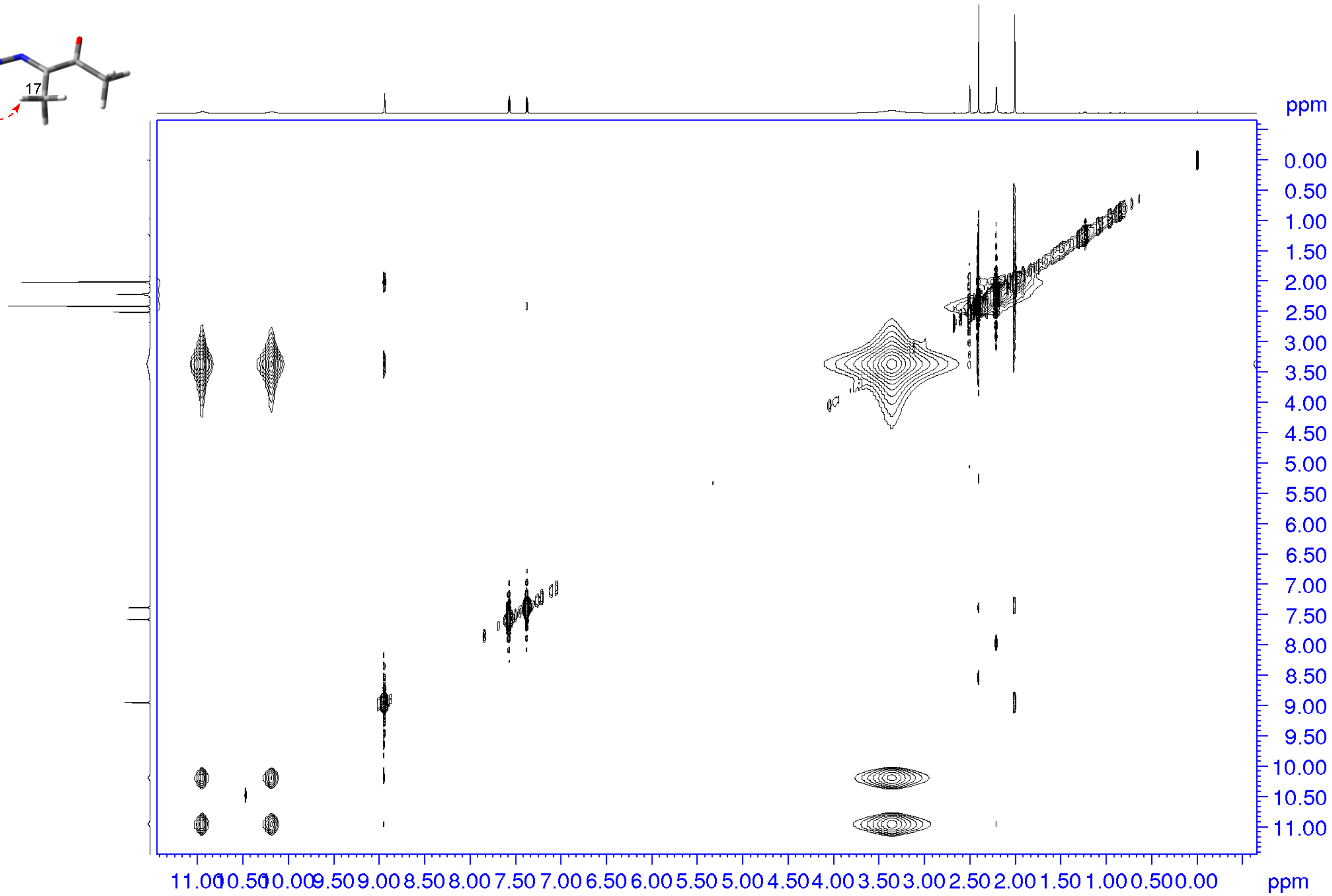

Figure S4. Spectral data for penzonemycin B (**2**).

(A) HR-ESI-MS

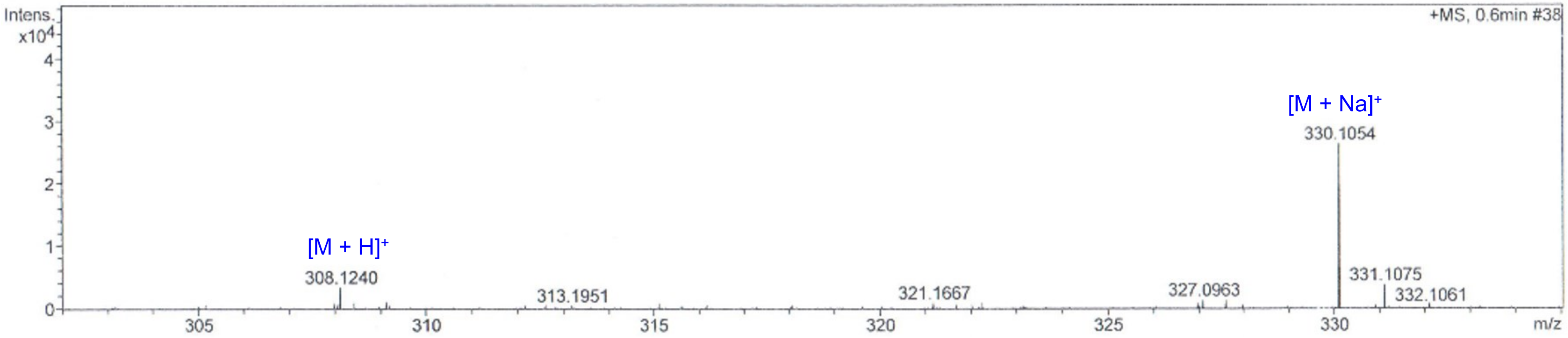

(B) IR

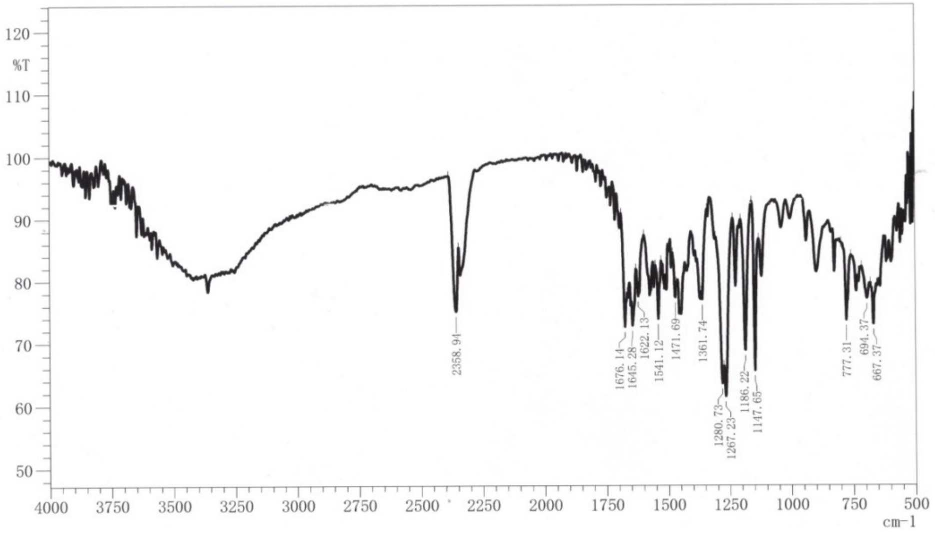

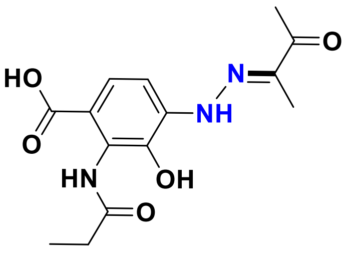

2

Chemical Formula: $C_{14}H_{17}N_3O_5$

caculated for $[M + H]^+$: 308.1241

caculated for $[M + Na]^+$: 330.1060

Figure S4. Spectral data for penzonemycin B (**2**) (continued).

(C) The ^{1}H NMR spectrum of penzonemycin B (**2**) in DMSO-d_6.

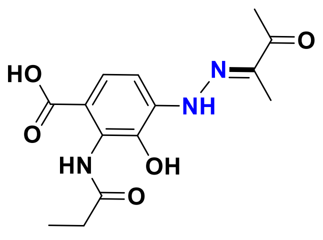

2

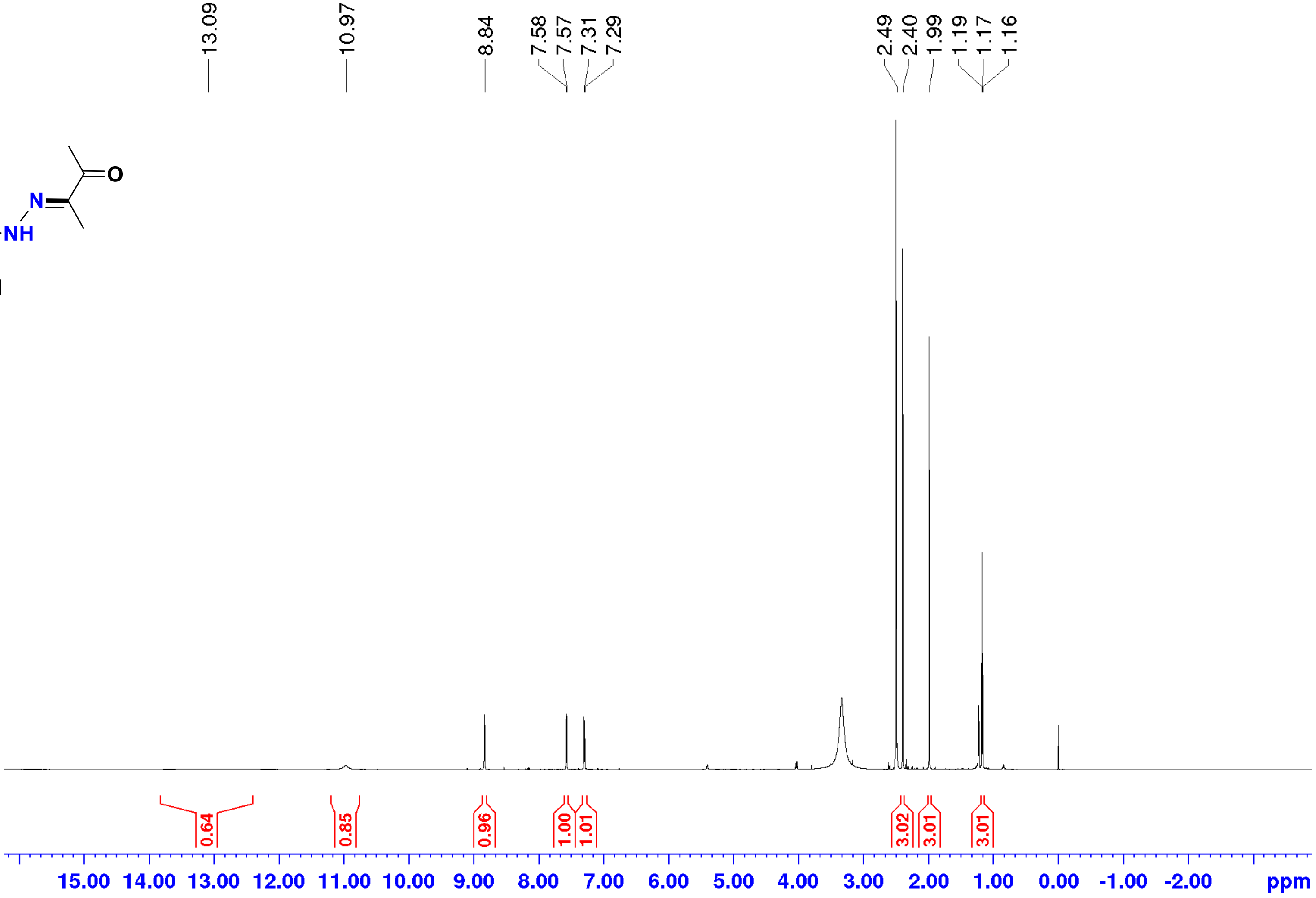

Figure S4. Spectral data for penzonemycin B (**2**) (continued).

(D) The ^{13}C NMR spectra of penzonemycin B (**2**) in DMSO-d_6.

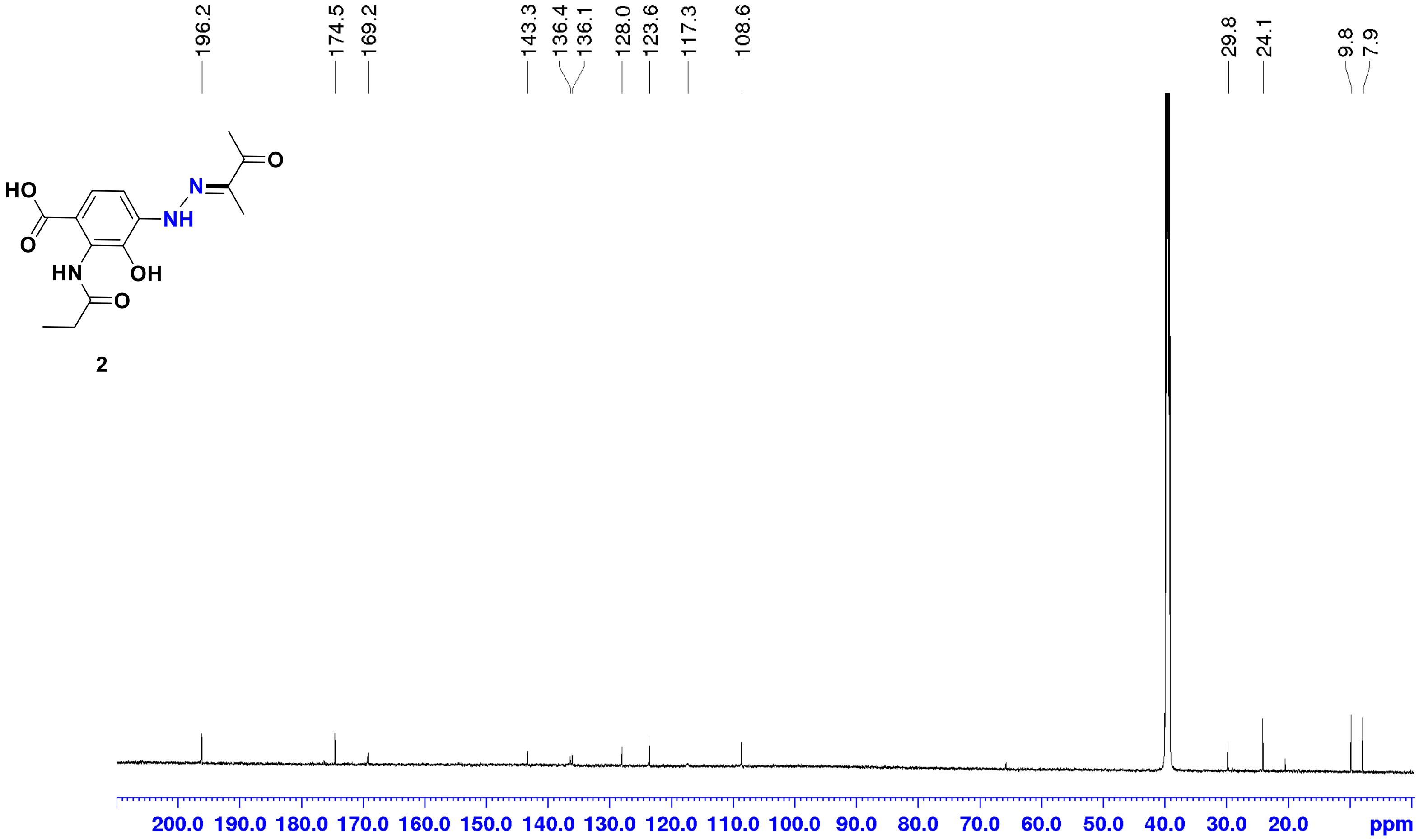

Figure S4. Spectral data for penzonemycin B (**2**) (continued).

(E) The HSQC spectrum of penzonemycin B (**2**) in DMSO-d_6.

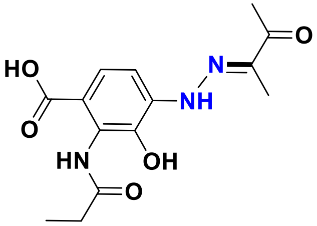

2

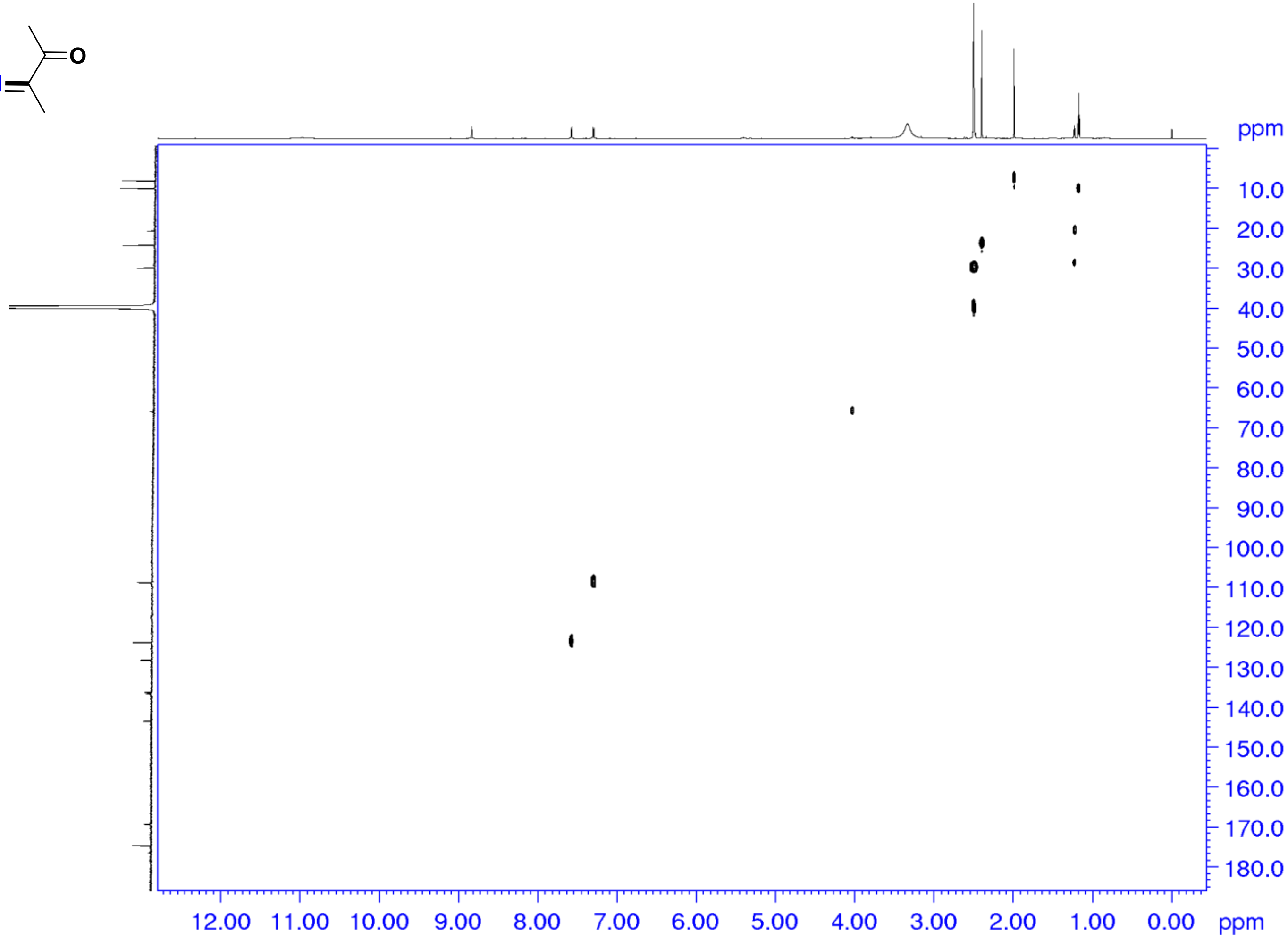

Figure S4. Spectral data for penzonemycin B (**2**) (continued).
(F) The HMBC spectrum of penzonemycin B (**2**) in DMSO-d_6.

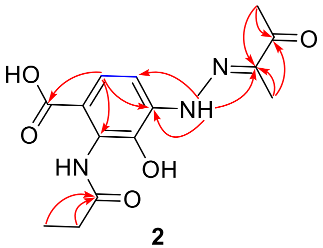

2

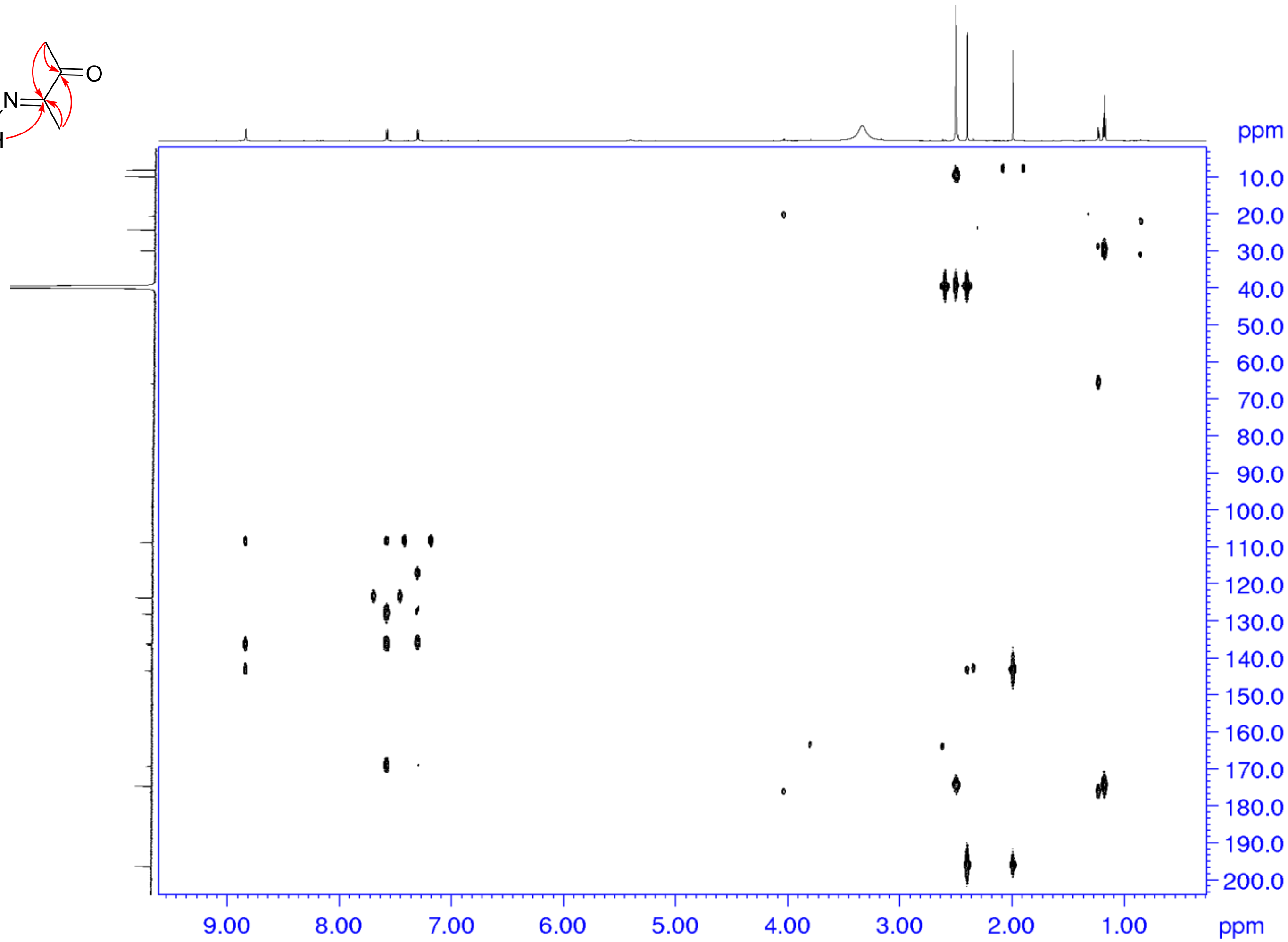

Figure S4. Spectral data for penzonemycin B (**2**) (continued).

(G) The ^{1}H-^{1}H COSY spectrum of penzonemycin B (**2**) in DMSO-d_6.

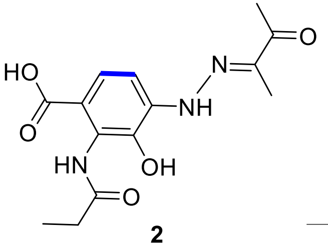

2

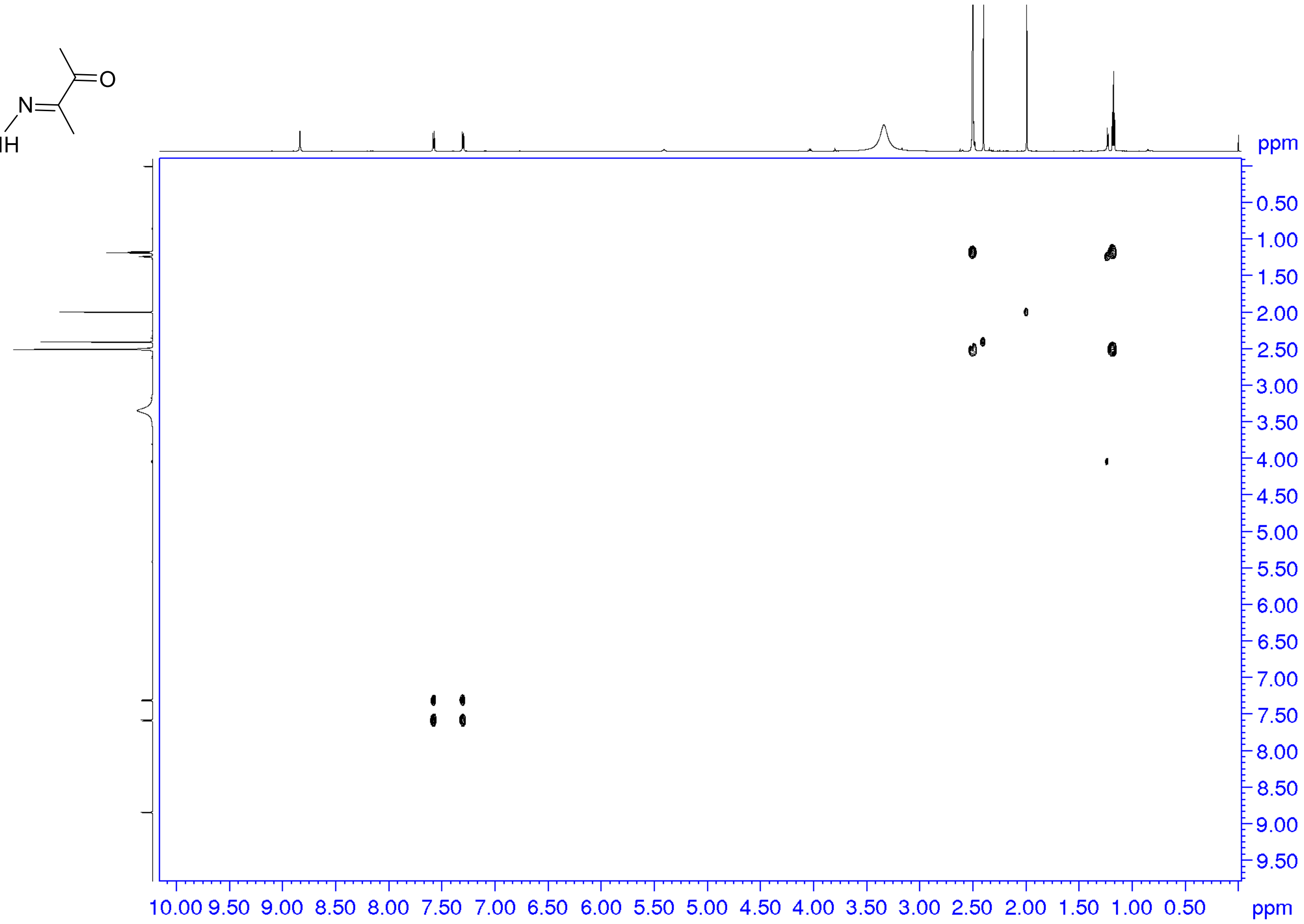

Figure S4. Spectral data for penzonemycin B (**2**) (continued).

(H) The NOESY spectrum of penzonemycin B (**2**) in DMSO-d_6.

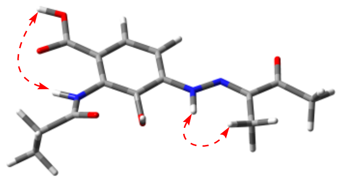

2

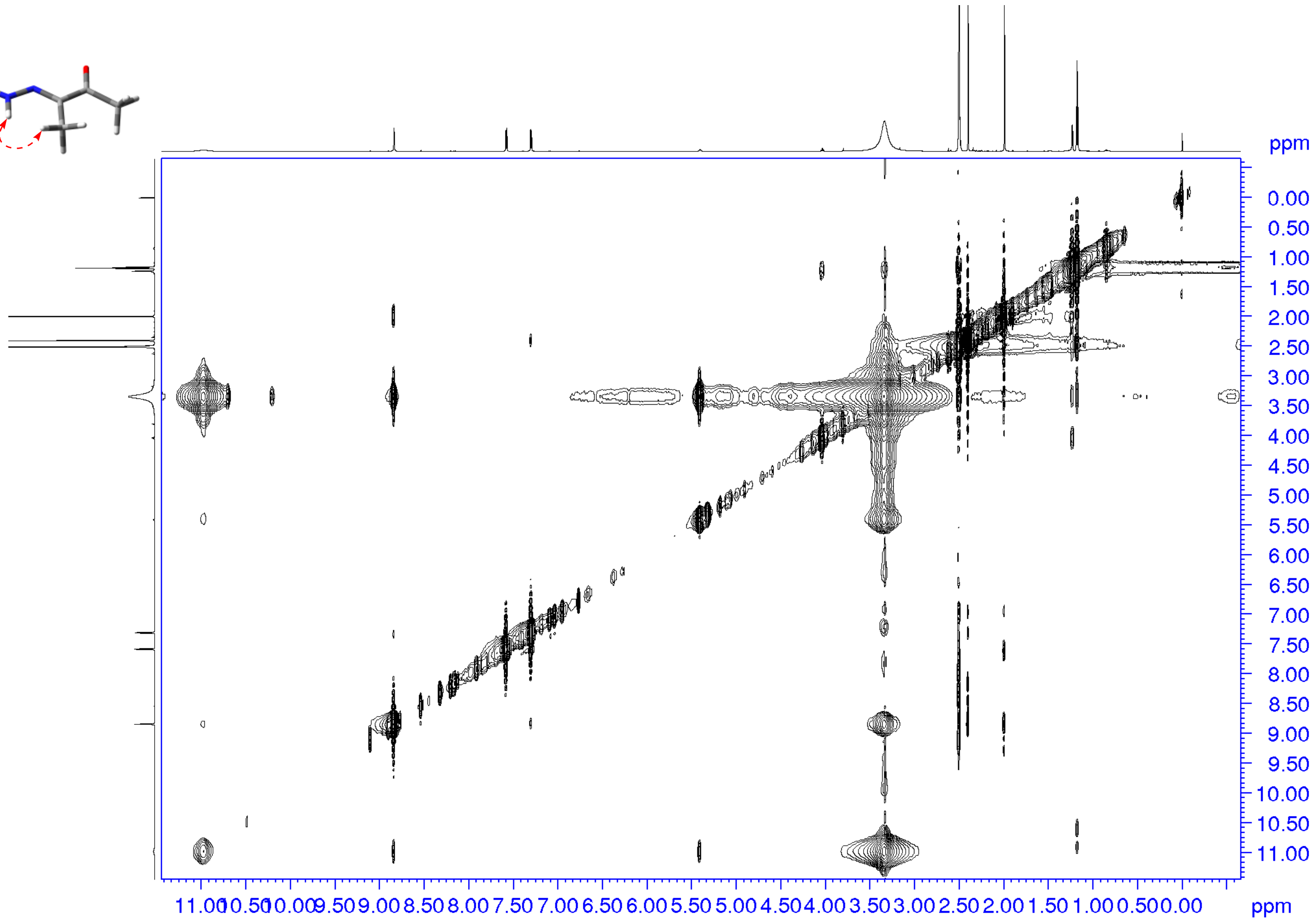

Figure S5. Spectral data for demethylmycemycin A (**3**).

(A) HRESIMS

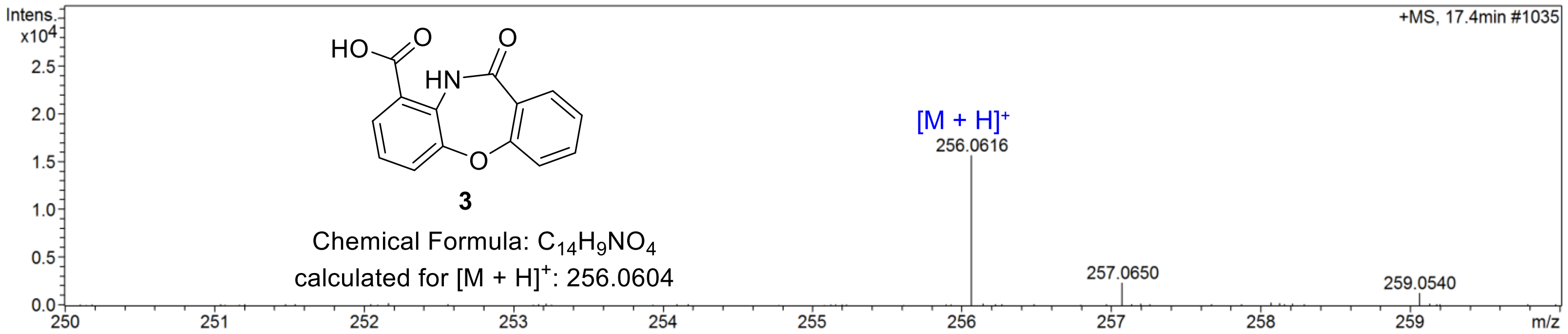

Meas. m/z	#	Formula	Score	m/z	err [mDa]	err [ppm]	mSigma	rdb	ej¥Conf	N-Rule
256.0616	1	C 14 H 10 N O 4	100.00	256.0604	-1.1	-4.4	11.8	10.5	even	ok

(B)

(**B**) UV

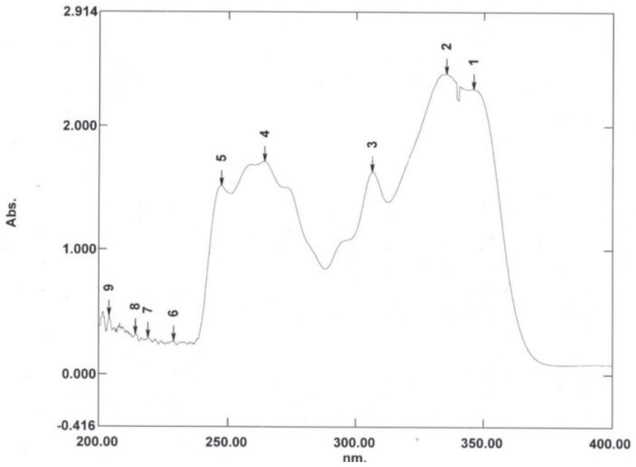

(**C**) IR

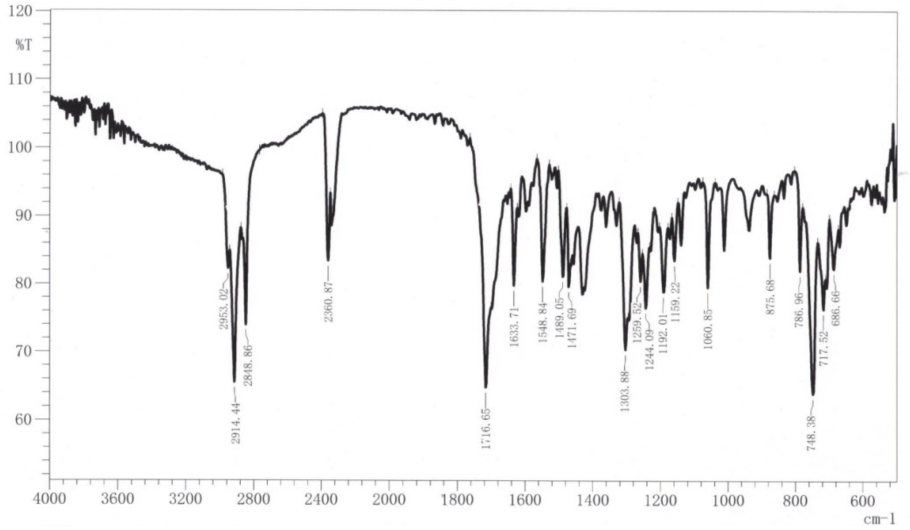

Figure S5. Spectral data for demethylmycemycin A (**3**) (continued).

(D) The ^{1}H NMR spectrum of demethylmycemycin A (**3**) in DMSO-d_6.

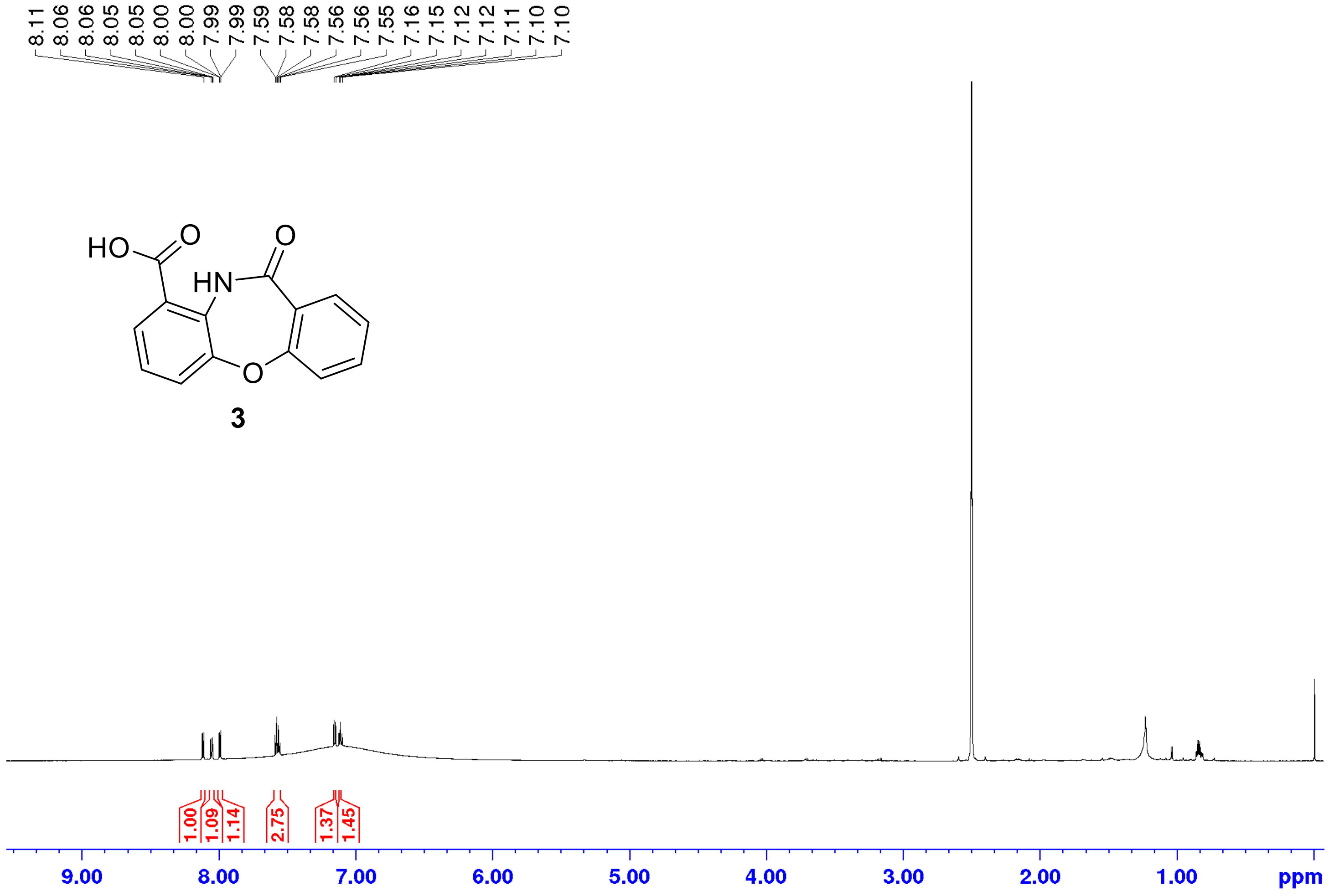

Figure S5. Spectral data for demethylmycemycin A (**3**) (continued).

(E) The ^{13}C NMR and DEPT135 spectra of demethylmycemycin A (**3**) in DMSO-d_6.

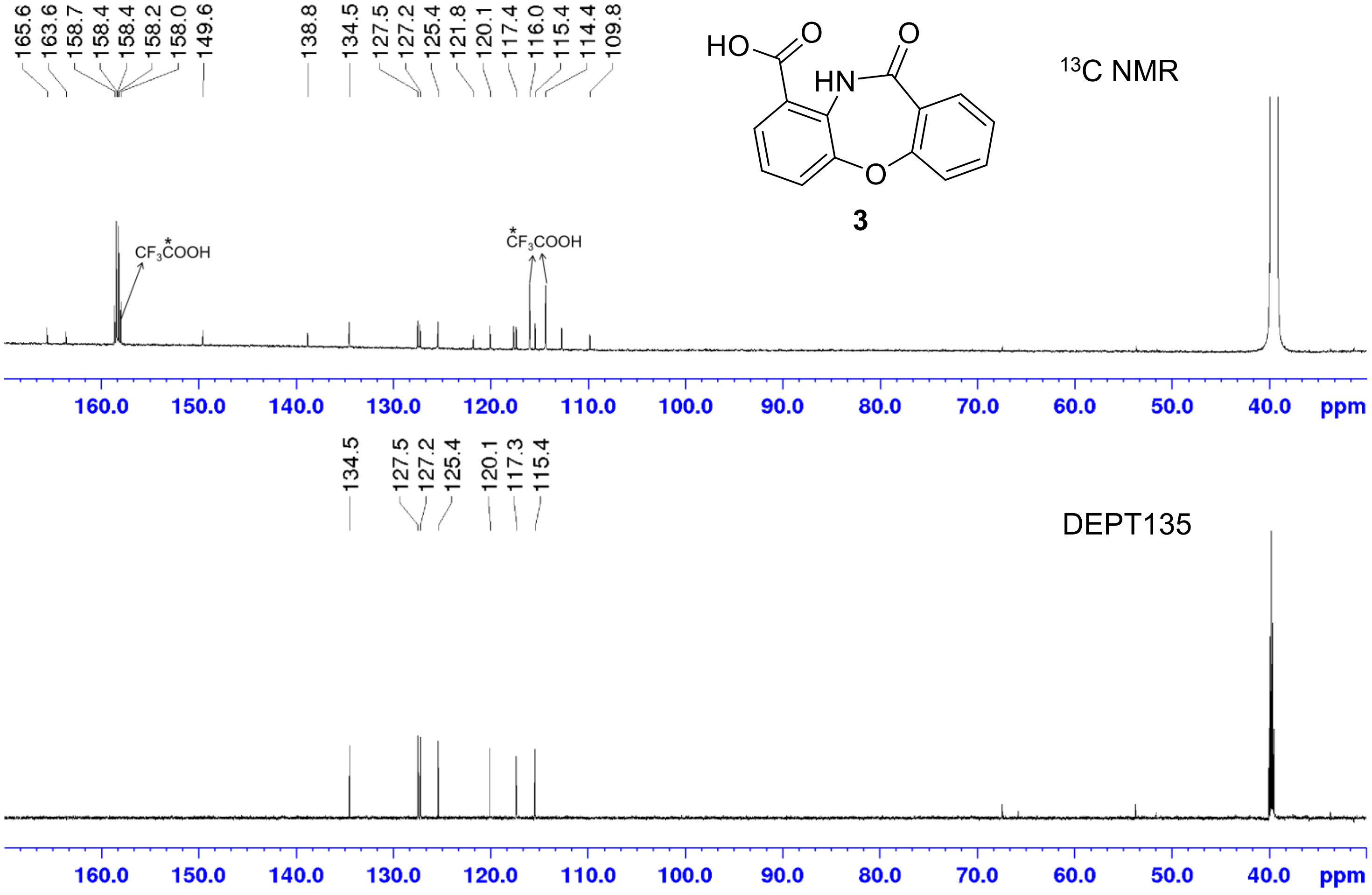

Figure S5. Spectral data for demethylmycemycin A (**3**) (continued).

(F) The HSQC spectrum of demethylmycemycin A (**3**) in DMSO-d_6.

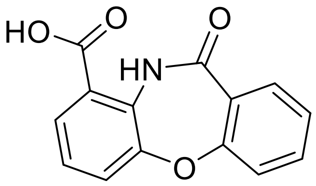

3

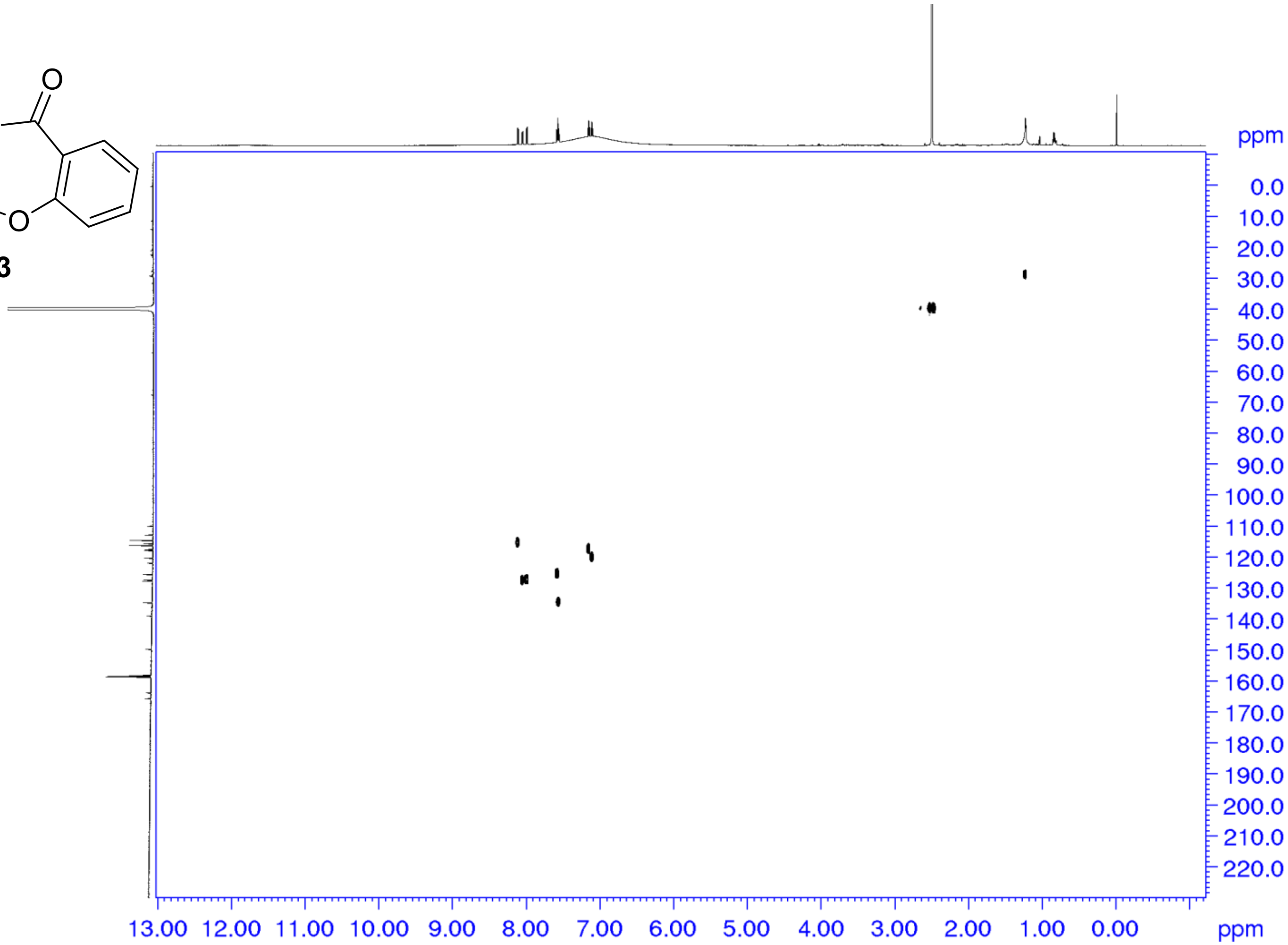

Figure S5. Spectral data for demethylmycemycin A (**3**) (continued).
(G) The HMBC spectrum of demethylmycemycin A (**3**) in DMSO-d_6.

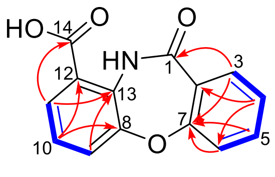

3

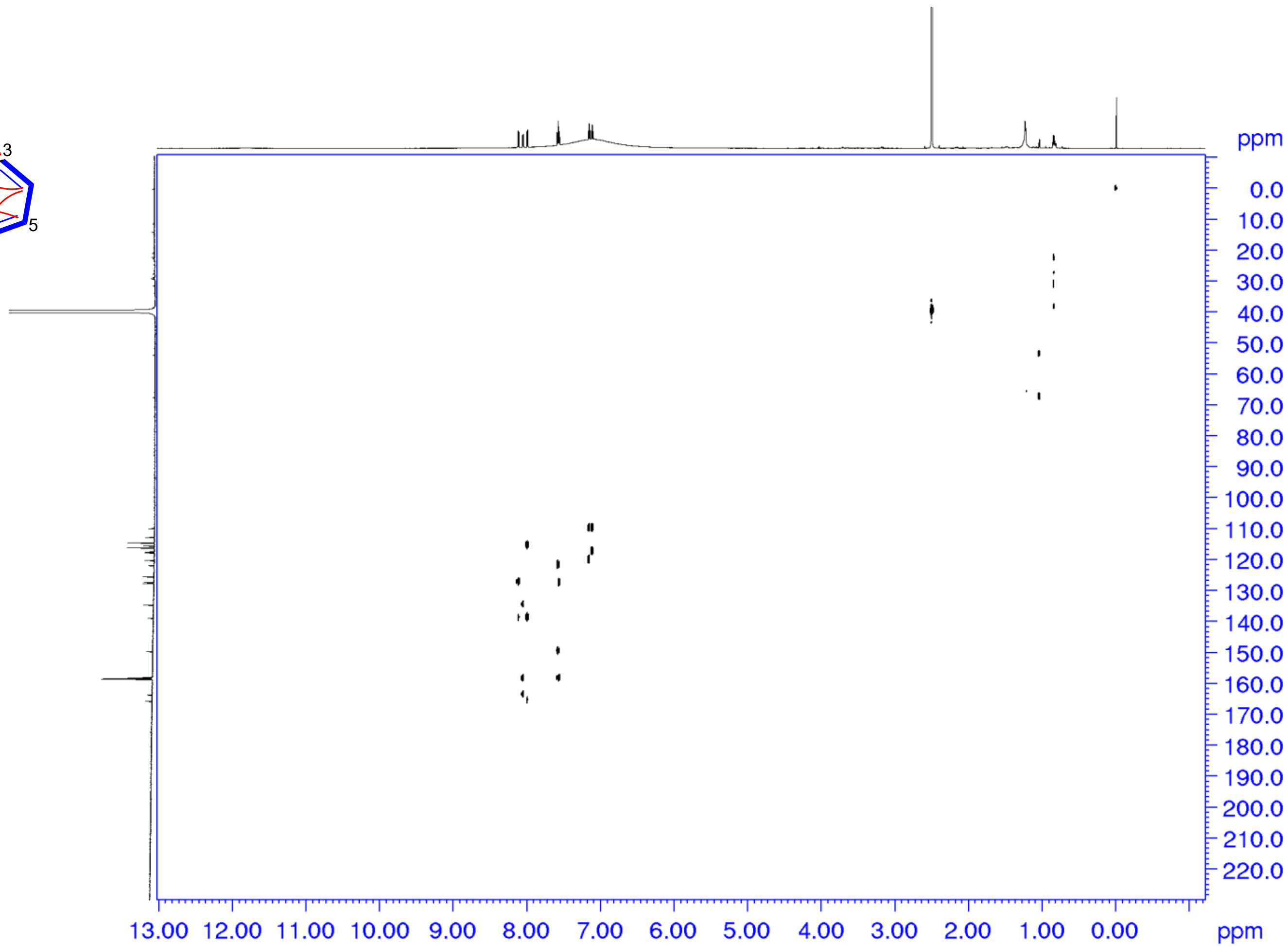

Figure S5. Spectral data for demethylmycemycin A (**3**) (continued).

(H) The ^{1}H-^{1}H COSY spectrum of demethylmycemycin A (**3**) in DMSO-d_6.

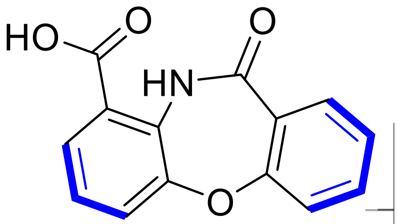

3

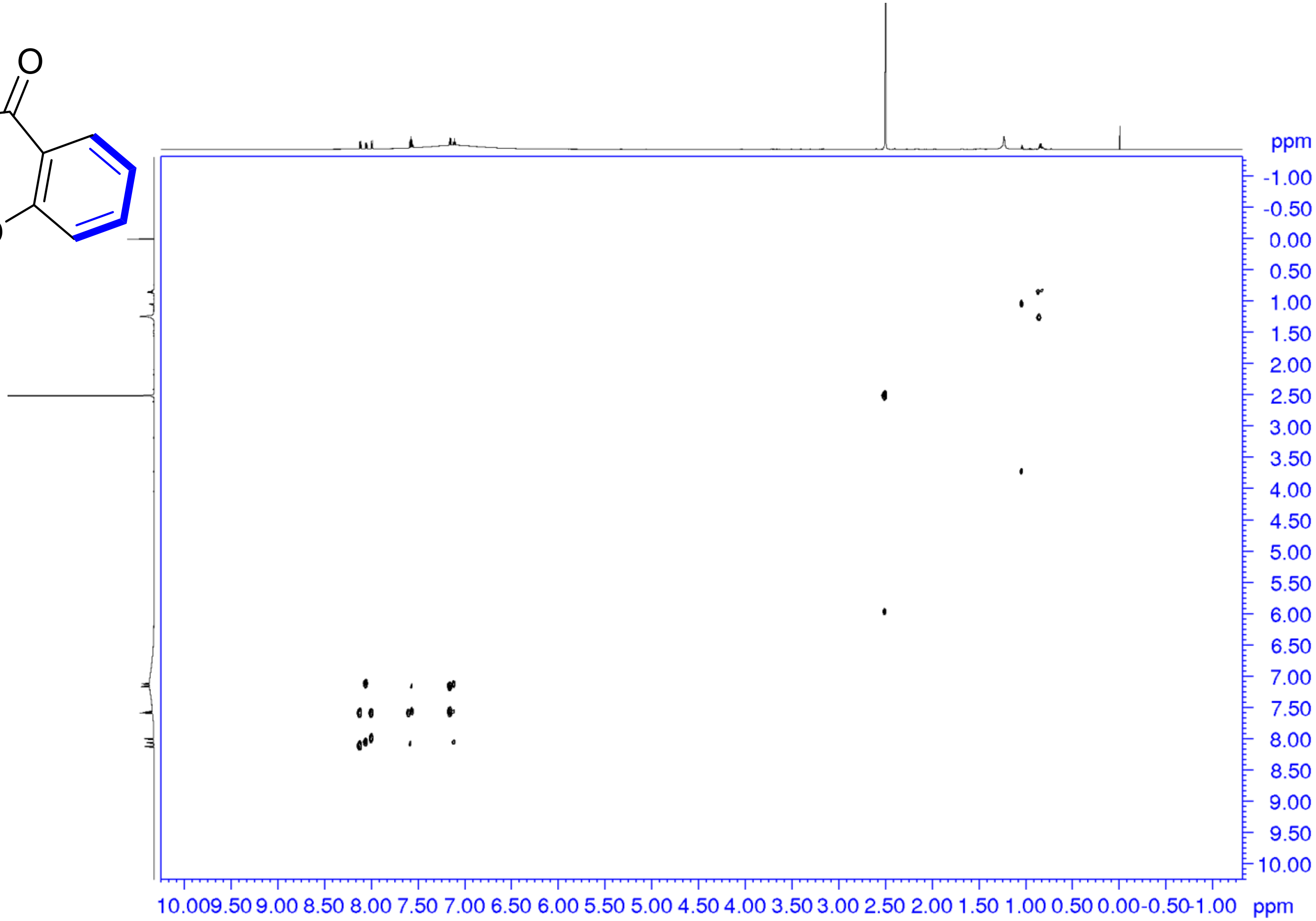

Figure S6. Spectral data for mycemycin A (**4**).

(A) The ^{1}H NMR spectrum of mycemycin A (**4**) in $CDCl_3$.

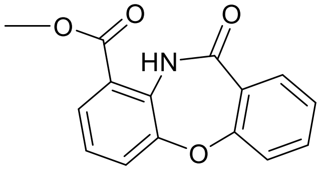

4

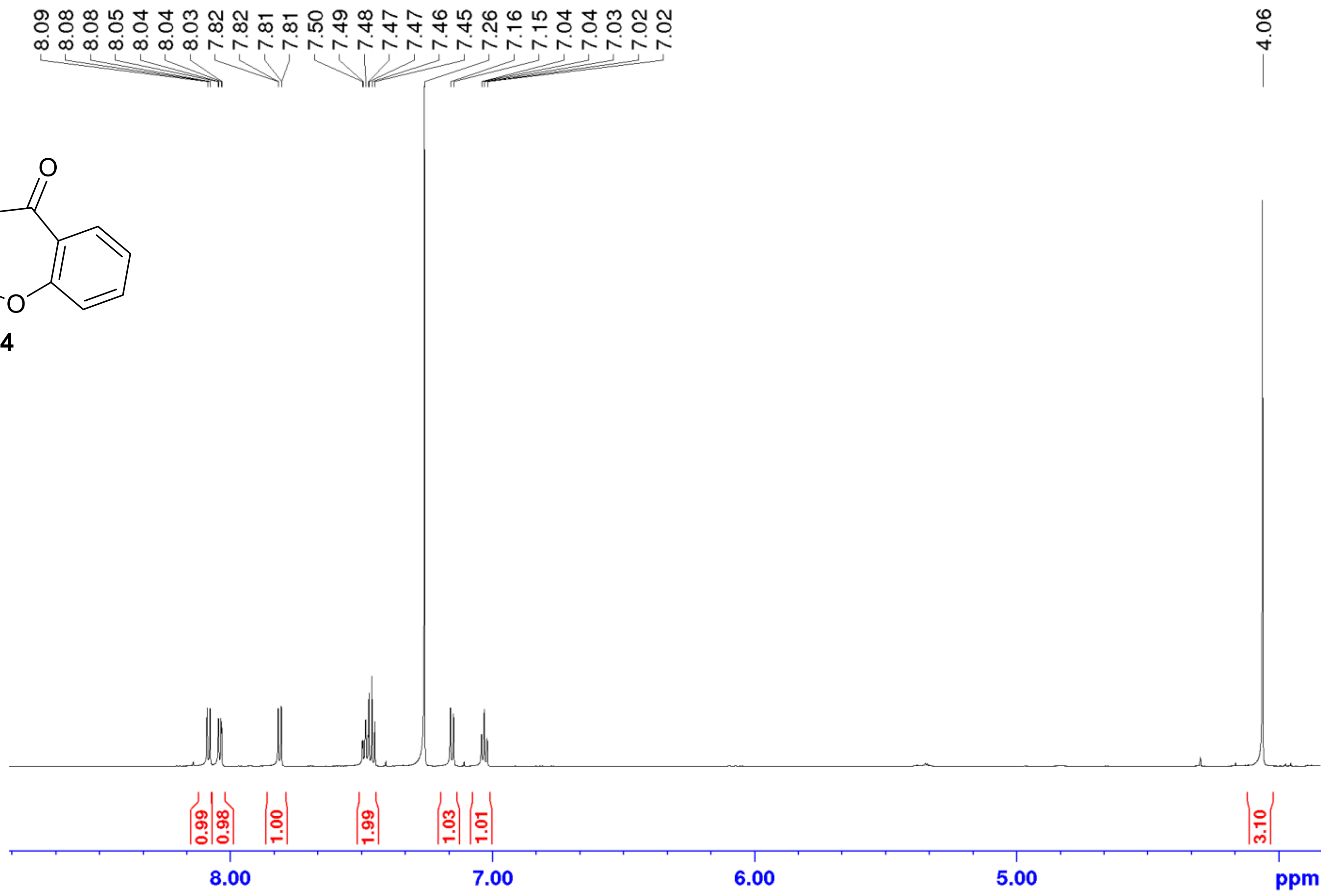

Figure S6. Spectral data for mycemycin A (**4**) (continued).

(B) The ^{13}C NMR spectrum of mycemycin A (**4**) in $CDCl_3$.

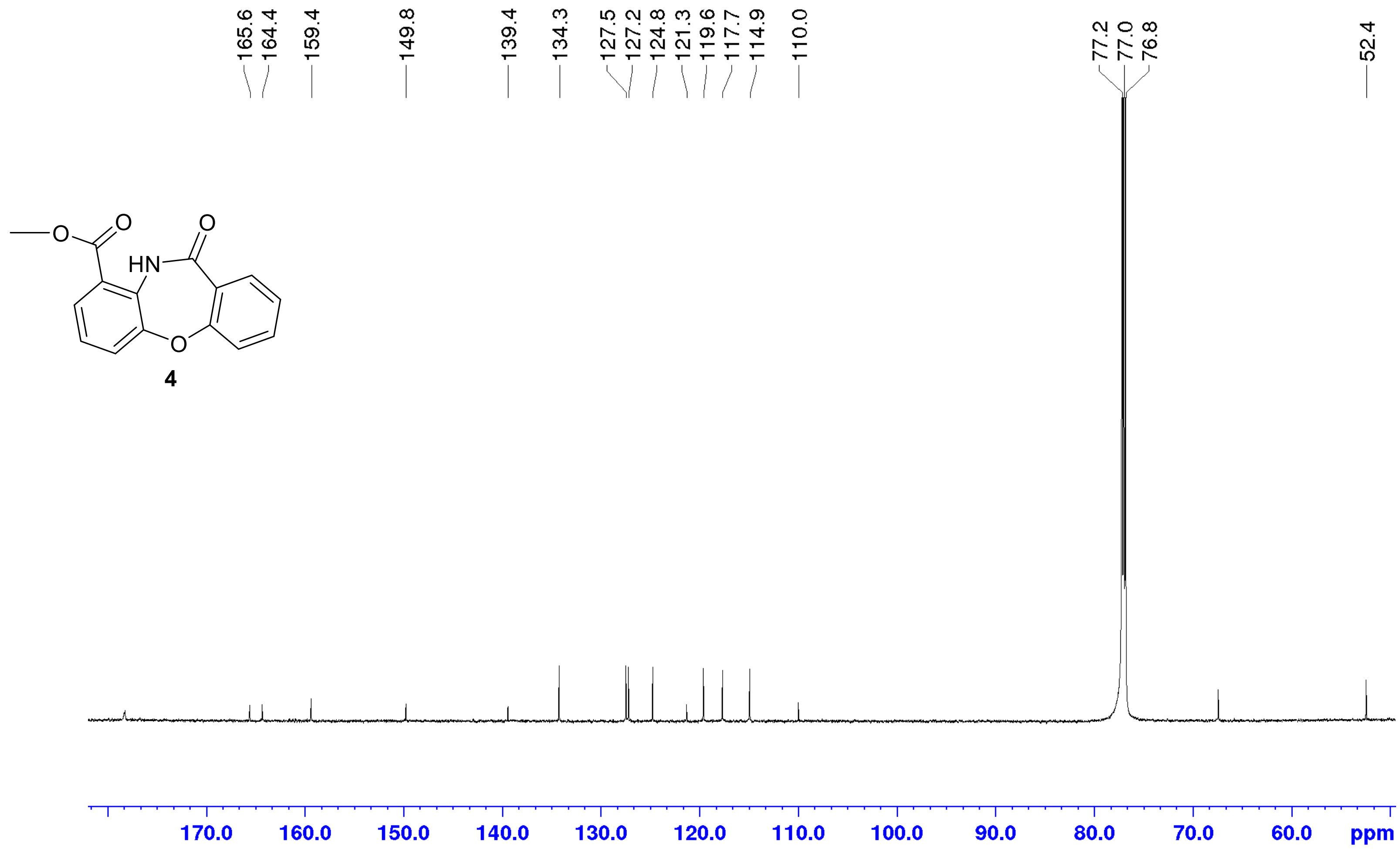

Figure S7. Spectral data for 9(*R*)-ganodermanol B (**5**).

(A) The ^{1}H NMR spectrum of 9(*R*)-ganodermanol B (**5**) in $CDCl_3$.

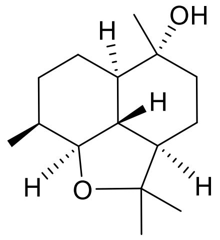

5

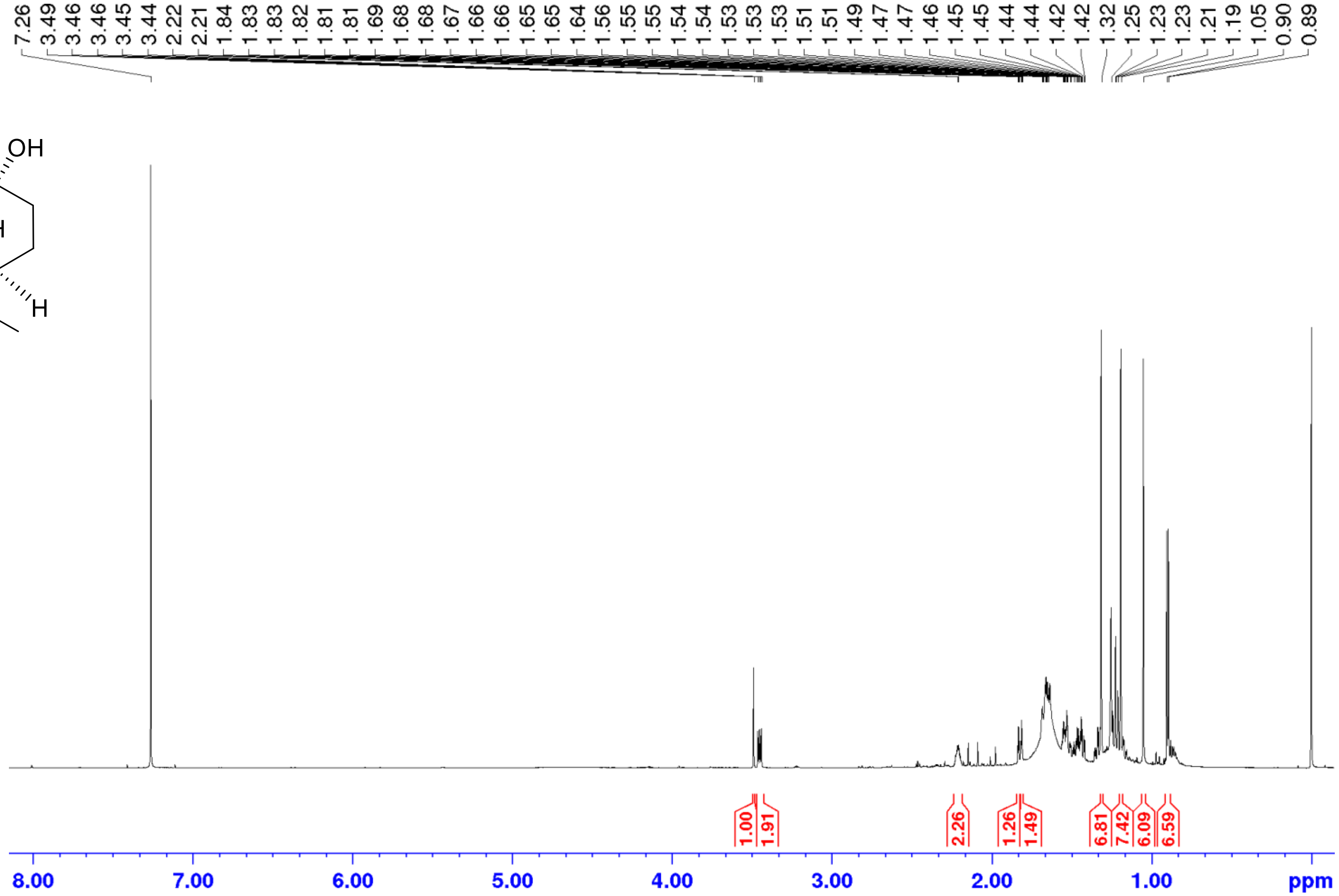

Figure S7. Spectral data for 9(*R*)-ganodermanol B (**5**) (continued).

(B) The ^{13}C NMR spectrum of 9(*R*)-ganodermanol B (**5**) in $CDCl_3$.

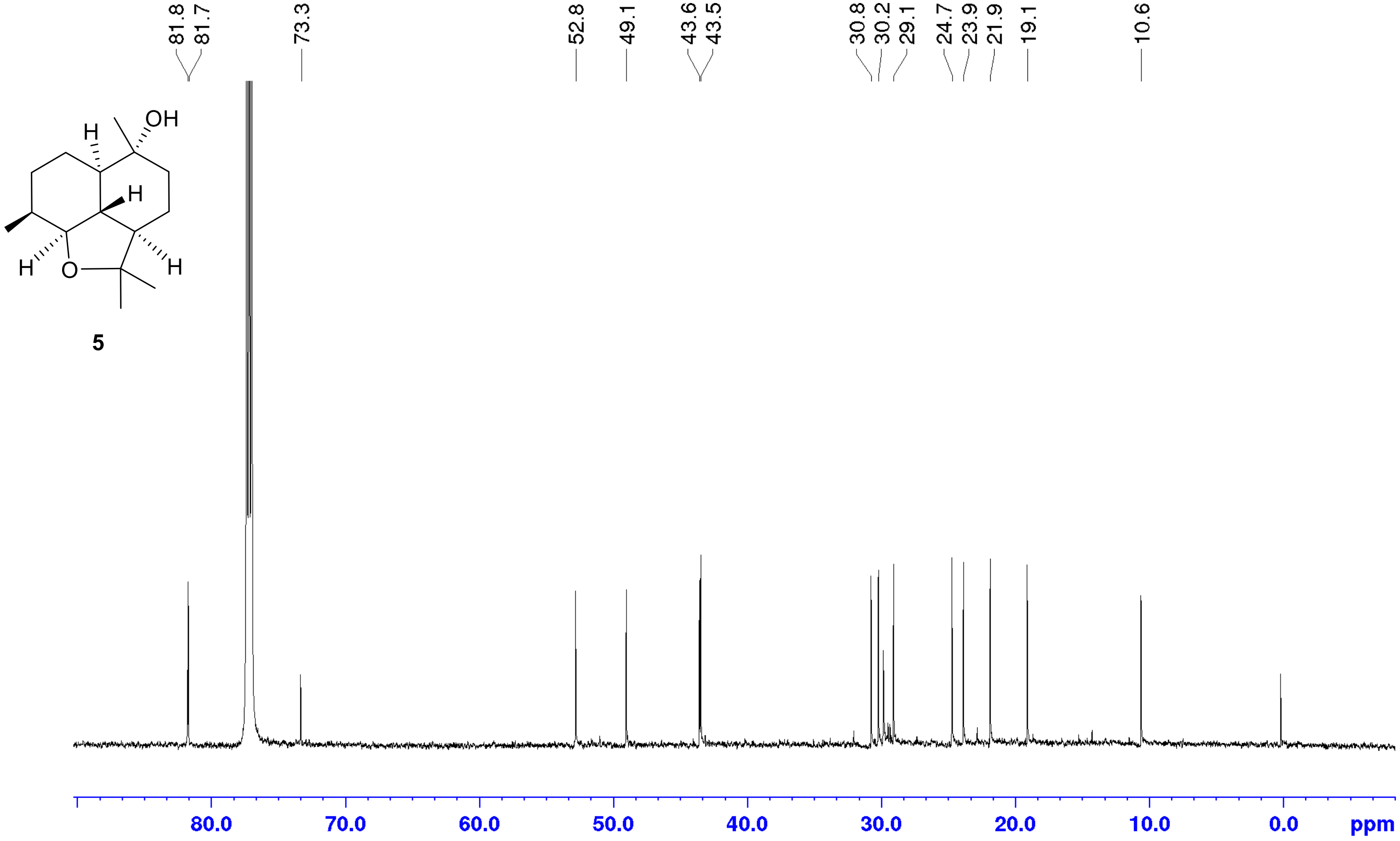

Figure S8. Spectral data for isopterocarpolone (**6**).

(A) The ^{1}H NMR spectrum of isopterocarpolone (**6**) in DMSO-d_6.

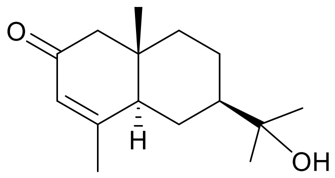

6

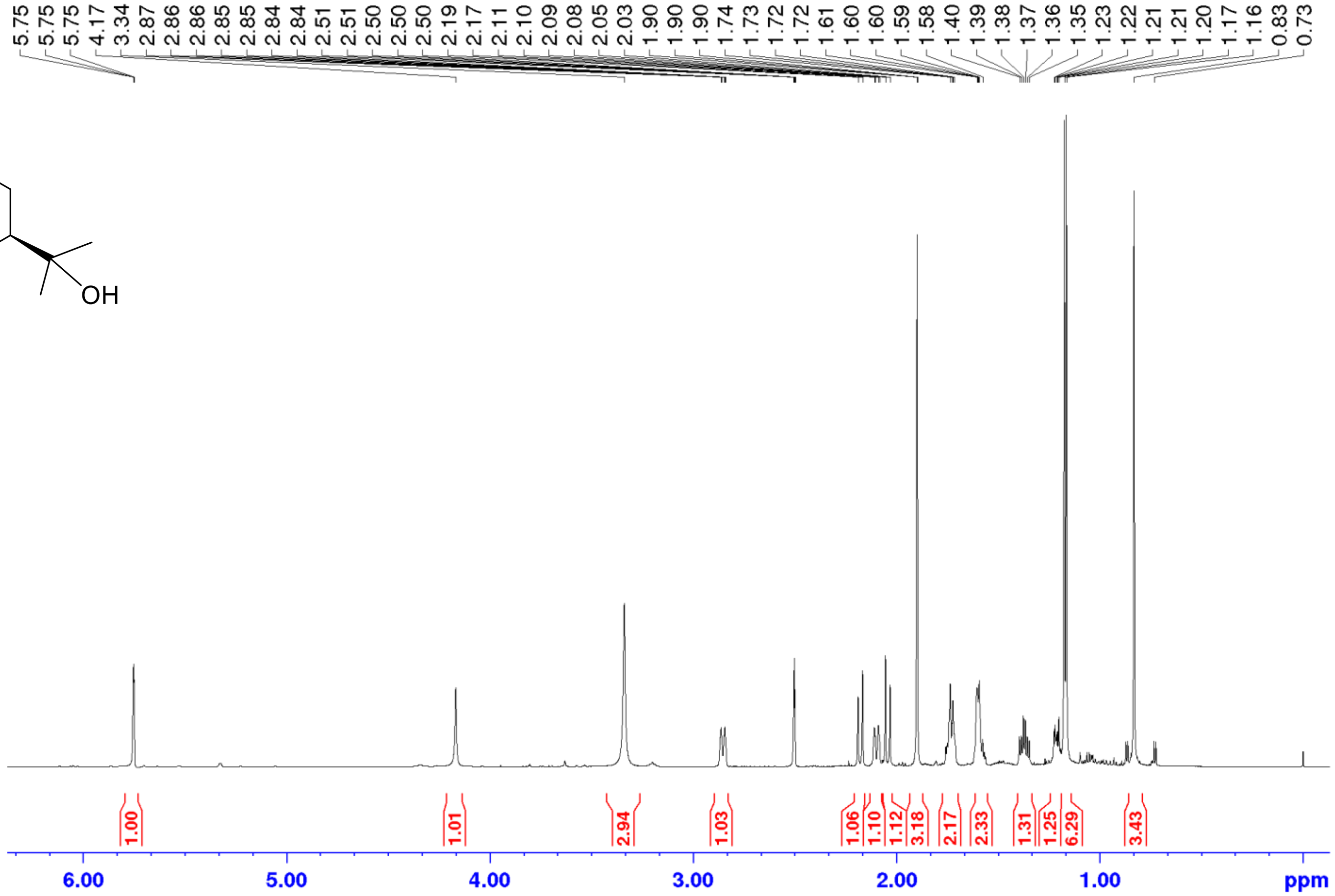

Figure S8. Spectral data for isopterocarpolone (**6**) (continued).

(B) The ^{13}C NMR spectrum of isopterocarpolone (**6**) in DMSO-d_6.

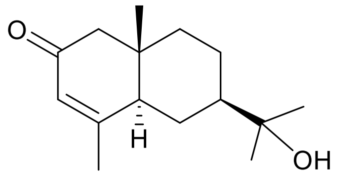

6

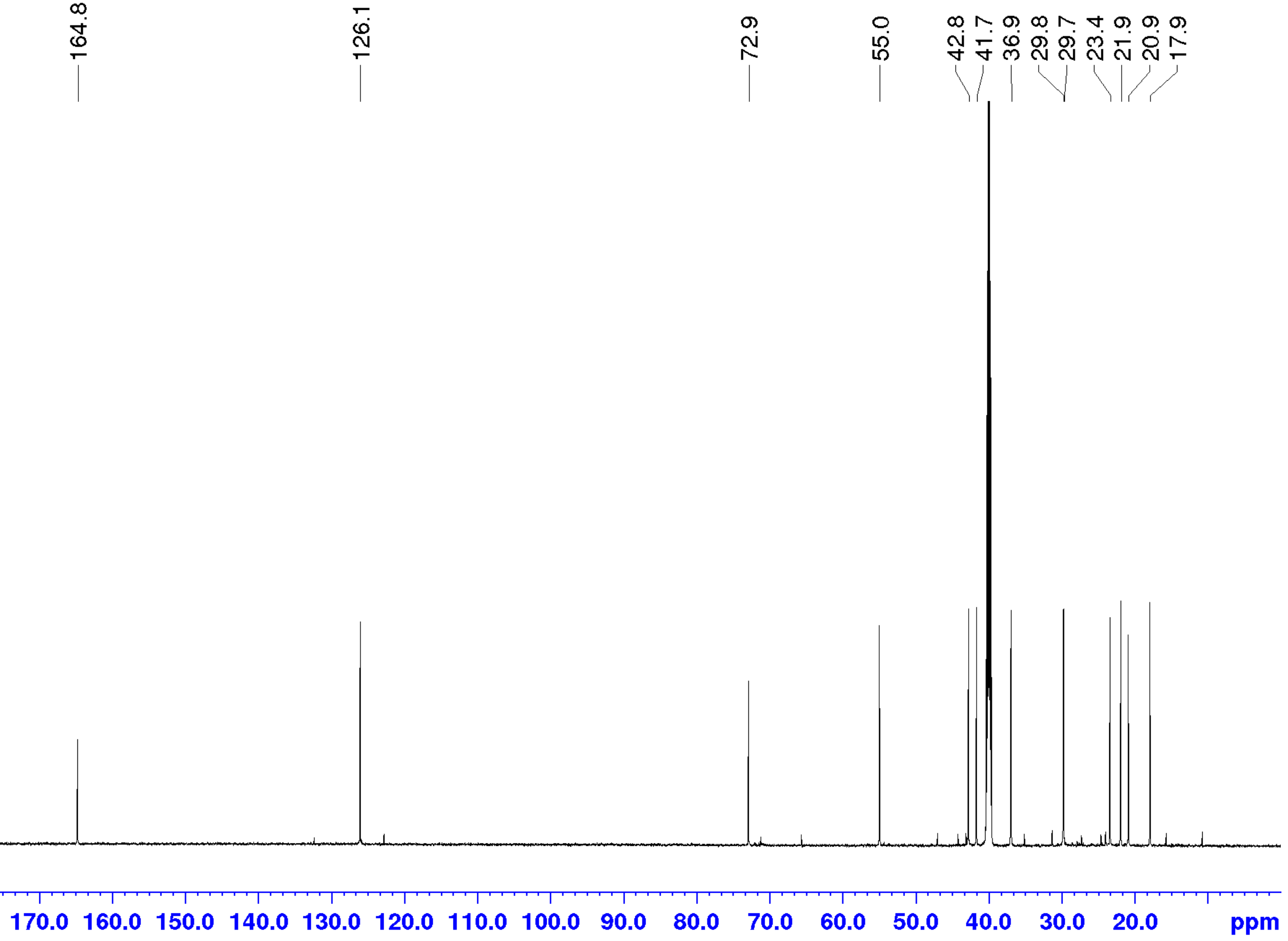

Supplement: Supplementary file 1 [file marinedrugs-20-00449-s001.zip › marinedrugs-1792552-supplementary.pdf]
